# Fluctuation-Response Design Rules for Nonequilibrium Flows

**Published:** 2026-02-27

**Authors:** Ying-Jen Yang, Ken A. Dill

**Affiliations:** Laufer Center of Physical and Quantitative Biology, Stony Brook University; Laufer Center of Physical and Quantitative Biology, Stony Brook University; Department of Physics, Stony Brook University; Department of Chemistry, Stony Brook University

## Abstract

Biological machines like molecular motors and enzymes operate in dynamic cycles representable as stochastic flows on networks. Current stochastic dynamics describes such flows on fixed networks. Here, we develop a scalable approach to *network design* in which local transition rates can be systematically varied to achieve global dynamical objectives. It is based on the fluctuation-response duality in the recent Caliber Force Theory—a pathentropy variational formalism for nonequilibria. This approach scales efficiently with network complexity and gives new insights, for example revealing the transition from timing- to branching-dominated fluctuations in a kinesin motor model.

## Introduction:

Consider a stochastic dynamical process transitioning among a network of states. In biophysics alone, such stochastic network flows are involved in the molecular mechanisms of motors and pumps [1–3], ultrasensitive switches in flagella [4, 5], the catalytic and allosteric actions in enzymes [6–9], phosphorylation cycles [10, 11], energy and chemical transduction [12, 13] and others. A fundamental goal is to control the long-term statistics of flows, given the local transition rates.

While Markov State Models and Master Equations describe these dynamics [14], they do not prescribe design. What is missing are principles for the optimization, control, and evolution of network flows: how should one systematically tune local transition rates to achieve specific global functions? Mathematically, this requires solving the response gradient—knowing exactly how local perturbations shift the long-term stochastic dynamics. However, raw gradients evaluated case-by-case do not reveal general principles. To navigate systematically, we exploit a key physical connection: general response relations are encoded in the system’s fluctuations.

Echoing Onsager’s regression hypothesis, recent nonequilibrium (NEQ) advances have established diverse fluctuation-response relations—equalities [15–20] or bounds [17, 20–23]—revealing that “noisier” mechanisms are inherently more susceptible to perturbative control. However, operationalizing this for NEQ flow design has some obstacles. First, design demands gradients, not just bounds. While performance limits are insightful [17, 20–23], inequalities inherently lack the local directional information needed to navigate parameter space. Second, we need to design and control the flux statistics too, not just the average state occupancy or the mean fluxes. Response relations on state occupancy [15, 22] do not control flux fluctuations. Third, scalability is paramount. Standard techniques, such as differentiating generating functions [24, 25] or summing over spanning trees [12, 14, 17, 21], offer physical insights but scale unfavorably with system size, rendering large-network optimization computationally demanding. We need an actionable, complete and scalable framework.

Here, we address this need by establishing a principle-based design framework rooted in Caliber Force Theory (CFT) [26]. By projecting the independent transition noise [19] onto the orthogonal counting observables in CFT, we show that kinetic fluctuation-response relations originate from the theory’s fundamental observable-force conjugacy. This structure is encoded in a Jacobian matrix linking transition rates to *forces*. This underpinning provides a systematic and scalable design framework. Applied to a data-fitted kinesin model [2], it parses fluctuations to reveal the chemomechanical covariances underlying a load-dependent noise transition. Computationally, our framework overcomes the scalability bottleneck in evaluating gradients for the means and variances of fluxes in large networks. Analytically, it unifies NEQ response relations, extending previous results [17, 18, 20] and deriving kinetic bounds reflecting population depletion and Le Chatelier-like compensation. This framework transforms the geometry of spontaneous fluctuations into a scalable “road-map” for gradient-based design.

## Theoretical Framework:

Our foundation here is CFT, a dynamical variational formalism that mirrors the structure of equilibrium (EQ) thermodynamics [26]. We begin by considering the *path entropy* of Markov jump processes—defined as the logarithm of path probability ratio $\ln\left(P_{k}/P_{u}\right)$. It measures the path-wise entropic cost of driving a unit-rate reference process u to the dynamics with transition rate k. For a path $\omega_{t}$ with duration t, it can be expressed in terms of *extensive counting observables* [27]:

(1) $$\ln\frac{P_{k}\left(\omega_{t}\right)}{P_{u}\left(\omega_{t}\right)}=\sum_{i\neq j}N_{ij}\left(\omega_{t}\right)\ln k_{ij}-\sum_{i}T_{i}\left(\omega_{t}\right)\varepsilon_{i}(k),$$

where $N_{ij}$ is the number of transitions i→j, $T_{i}$ is the total dwell time of state i, and $\varepsilon_{i}=\sum_{j(\neq i)}\left(k_{ij}-1\right)$ is the excess escape rate relative to the unit-rate reference. However, these raw observables have undesirable redundancies. Conservation laws—time additivity $\sum_{i}T_{i}=t$ and Kirchhoff’s current balance $\sum_{j(\neq i)}N_{ij}\sim \sum_{j(\neq i)}N_{ji}$ [28]—enforce strong coupling among them in the long term, rendering the naive counts $\left(N_{ij},T_{i}\right)$ degenerate axes for control.

CFT resolves this redundancy by identifying the *asymptotically orthogonal* counting variables X and relating the path entropy with their conjugate forces F—defined as the path entropy derivatives [26, 27]:

(2) $$\ln\frac{P_{k}\left(\omega_{t}\right)}{P_{u}\left(\omega_{t}\right)}\sim F(k)\cdot X\left(\omega_{t}\right)-c(k)t$$

where the caliber (ct) resembles for our NEQ processes the role that the free energy plays for EQ processes. The vector $X=\left({\text{Φ}}_{ij},T_{n},{\text{Ψ}}_{c}\right)$ is a complete observable basis consistent with the asymptotic constraints: (i) **edge traffic** ${\text{Φ}}_{ij}=N_{ij}+N_{ji}$; (ii) **dwell times** $T_{n}$ (excluding a reference m); and (iii) **cycle net fluxes** ${\text{Ψ}}_{c}=N_{ab}-N_{ba}$, defined by the net flux across the chord ab of a fundamental cycle c (mnemonic: the time irreversible counterpart to traffic, Ψ≡“i”+Φ). Dividing by time gives their rates $x=\left({\text{ϕ}}_{ij},f_{n},\psi_{c}\right)$, and taking steady-state averages yields the intensive rate coordinate for the parameter space $\langle x\rangle =\langle X\rangle /t=\left(\tau_{ij},\pi_{n},J_{c}\right)$. Their conjugate forces F are the affinities to edge exchange, node dwelling, and cycle completion [26]:

(3) $$\begin{array}{l} F_{\text{edge},ij}=\frac{1}{2}\ln k_{ij}k_{ji},\\ F_{\text{node},n}=\sum_{i(\neq m)}\left(k_{mi}-1\right)-\sum_{j(\neq n)}\left(k_{nj}-1\right),\\ F_{\text{cycle},c}=\frac{1}{2}\ln\frac{k_{i_{0}i_{1}}k_{i_{1}i_{2}}\cdots k_{i_{\sigma }i_{0}}}{k_{i_{0}i_{\sigma }}k_{i_{\sigma }i_{\sigma -1}}\cdots k_{i_{1}i_{0}}} \end{array}$$

where $i_{0}i_{1}i_{2}\ldots i_{\sigma }i_{0}$ is the state sequence of the cycle c. Crucially, the caliber rate $c(k)=\sum_{j\neq m}\left(k_{mj}-1\right)$ functions as the log dynamic partition function that generates observable statistics when parameterized by the forces F [26, 27].

The asymptotic form of Eq. (2) establishes fundamental *conjugate relations* between the observables and the forces. This conjugate structure dictates a fundamental duality between *observable fluctuations* and their corresponding *force responses*: the average susceptibility of the CFT rate observables $x_{\alpha }$ to a force $F_{\beta }$—while holding all other forces fixed—is exactly the asymptotic covariance [26]:

(4) $${\left.\frac{\partial \langle x_{\alpha }\rangle }{\partial F_{\beta }}\right|}_{F_{\gamma \neq \beta }}=\frac{\partial^{2}c(F)}{\partial F_{\alpha }\;\partial F_{\beta }}=\underset{t\to \infty }{\lim}t\;\text{Cov}\left[x_{\alpha },x_{\beta }\right].$$

Thus, fluctuations equal susceptibilities: (a) the strictly positive variance implies *monotonic* force responses: just as heat capacity is positive, any average rate observable $\langle x_{\alpha }\rangle \in \left(\tau_{ij},\pi_{n},J_{c}\right)$ must increase with its own conjugate drive $F_{\alpha }$; (b) the symmetry of covariance enforces a *generalized Maxwell-Onsager reciprocity* far from equilibrium: the susceptibility of observable $\langle x_{\alpha }\rangle$ to force $F_{\beta }$ is identical to the response of $\langle x_{\beta }\rangle$ to force $F_{\alpha }$.

While the forces F have clear physical meanings as affinities, practical control more often involves tuning specific transition rates $k_{ij}$ while fixing others, rather than manipulating one force while clamping the rest. We thus map the force-response conjugacy in CFT to the rate-response landscape:

(5) $${\left.\frac{\partial \langle x\rangle }{\partial \ln k_{ij}}\right|}_{k_{ab},\;ab\neq ij}=\sum_{\beta }\frac{\partial \langle x\rangle }{\partial F_{\beta }}\frac{\partial F_{\beta }}{\partial \ln k_{ij}}$$

**Figure 1.**
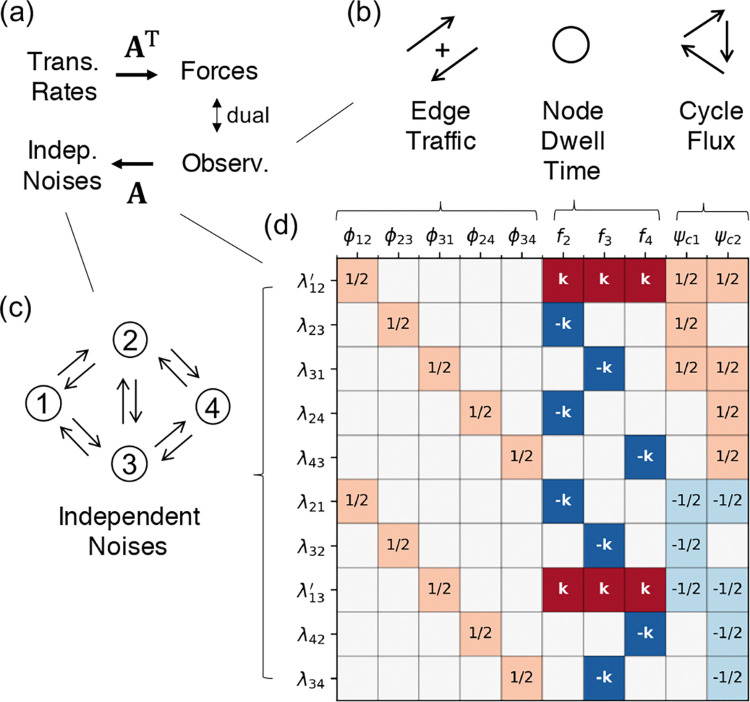
The sparse structure of fluctuation-response duality. (**a**) The Jacobian matrix A acts as the bridge between stochasticity and control: it maps physical observables x to independent noise sources λ (Fluctuation Geometry), while simultaneously linking transition rates lnk to conjugate forces F (Response Geometry). (**b**) The complete observable basis: edge traffic $\phi_{ij}$, node frequency $f_{n}$, and cycle net flux $\psi_{c}$. (**c**) A representative 4-state network showing that every transition edge generates an intrinsic independent noise source $\lambda_{ij}$. (**d**) The explicit structure of the Jacobian A for the 4-state example. The symbol k (or −k) denotes the transition rate $k_{ij}$ corresponding to the specific row index. The prime notation $\lambda_{mj}^{\prime }$ denotes the shifted noise source $\lambda_{mj}^{\prime }=\lambda_{mj}+k_{mj}$ for transitions leaving the reference state m=1. White squares denote structural zeros.

The projection is encoded by a sparse matrix $A_{(ij),\beta }\equiv \partial F_{\beta }/\partial \ln k_{ij}$, the Jacobian linking the transition rates k to the forces F [27], illustrated in Fig. 1.

To further operationalize this, we identify the independent noise sources, $\lambda_{ij}=\left(N_{ij}-k_{ij}T_{i}\right)/t$, in a Markov jump process, whose asymptotic covariances are diagonal [19]:

(6) $$t\cdot \text{Cov}\left[\lambda_{ij},\lambda_{mn}\right]\sim \pi_{i}k_{ij}\;\delta_{i,m}\;\delta_{j,n}.$$

These noise sources are asymptotically linearly spanned by the CFT observables via the same Jacobian A [27]:

(7) $$\lambda \left[\omega_{t}\right]\sim A(k)\;x\left[\omega_{t}\right]-\nabla_{\ln\;k}c(k).$$

Combining these yields the central **Response-Inverse-Matrix (RIM)** relation [27]:

(8) $$\frac{\partial \langle x_{\alpha }\rangle }{\partial \ln\;k_{ij}}=\underset{t\to \infty }{\lim}\;t\cdot \text{Cov}\left[x_{\alpha },\lambda_{ij}\right]\;\;=\pi_{i}k_{ij}{\left[A^{-1}\right]}_{\alpha ,(ij)}$$

where $A^{-1}=\nabla_{F}\;\ln\;k(F)$ is the Jacobian from the forces to the transition rates.

These equations constitute a *fluctuation-response duality* for rate perturbations, originating from the underlying *observable-force conjugacy*: the Jacobian A maps the CFT conjugates (x,F) onto the kinetic variables (λ, lnk). While the first equality in Eq. (8) recovers a known result recently highlighted by Zheng and Lu [19], the second equality reveals the underlying dual geometry, identifying responses explicitly as matrix elements of the inverse Jacobian $A^{-1}$. This gives three results: the linear mapping enables parsing fluctuations into independent components (Result 1); an algebraic closure of derivatives enables scalable gradient evaluation (Result 2); and Jacobian symmetries yield unified response relations, generalizing recent results [17, 18, 20] (Result 3).

### Fluctuation–Response Relations are the key to design and optimization.

While the statistical independence of noise sources λ is established [19], it is the linear mapping in Eq. (7) that identifies them as the complete, projectable coordinate axes. We exploit this geometric structure to decompose the total covariance between any two observables x, $x^{\prime }\in \left(\phi_{ij},f_{n},\psi_{c}\right)$ into a sum over their projections onto the orthonormal axes:

(9) $$\text{Cov}\left(x,x^{\prime }\right)\sim \sum_{i,j}\text{Cov}\left[x,{\hat{\lambda }}_{ij}\right]\cdot \text{Cov}\left[x^{\prime },{\hat{\lambda }}_{ij}\right],$$

where ${\hat{\lambda }}_{ij}=\lambda_{ij}/\sqrt{\pi_{i}k_{ij}/t}$ is the unit-variance noise source at transition i↦j. Geometrically, this is the inner product projection rule for vectors: $u\cdot v=\sum_{z}\left(u\cdot {\hat{e}}_{z}\right)\left(v\cdot {\hat{e}}_{z}\right)$. Crucially, the RIM relation (Eq. 8) further operationalizes this geometry. It transforms the covariance projections—which are otherwise difficult to derive from k—into simple matrix elements. Every term in the decomposition becomes explicitly evaluable via the inverse Jacobian $A^{-1}$.

We now apply Eq. (9) to a simple model of the kinesin molecular motor to illustrate how the motor “wastes time” through structural imprecision. We define a randomness parameter r: it is the variance-to-mean ratio of the net mechanical flux $\psi =\left(N_{+}-N_{-}\right)/t$, where $N_{\pm }$ denotes the forward and backward mechanical step counts. We express the motor’s randomness in terms of its susceptibility:

(10) $$\begin{array}{l} r=\underset{t\to \infty }{\lim}\frac{t\cdot \mathrm{Var}(\psi )}{\langle \psi \rangle }=\underset{t\to \infty }{\lim}\frac{1}{\langle \psi \rangle }\sum_{i,j}{\left(\text{Cov}\left[\psi ,{\hat{\lambda }}_{ij}\right]\right)}^{2}\\ \;=\frac{1}{\langle \psi \rangle }\sum_{i,j}\underset{\text{Sensitivity Contribution}}{\underset{︸}{\frac{k_{ij}}{\pi_{i}}{\left(\frac{\partial \langle \psi \rangle }{\partial k_{ij}}\right)}^{2}}}. \end{array}$$

This shows that imprecision couples to responsiveness: since the noise contribution scales with the squared sensitivity ${\left(\partial \langle \psi \rangle /\partial k\right)}^{2}$—a highly responsive step “amplifies” its own intrinsic noise into the overall variance. Crucially, this result moves beyond bounds like thermodynamic uncertainty relations [25, 30, 31] which constrain the *magnitude* of flux randomness based on global dynamical time irreversibility. Instead, Eq. (10) provides an *exact mechanistic decomposition* in terms of kinetic covariances and sensitivities. This approach directly identifies which specific molecular transitions act as the primary sources of the motor’s randomness, enabling the targeted dissection of the motor’s stochastic mechanism shown below.

**Figure 2.**
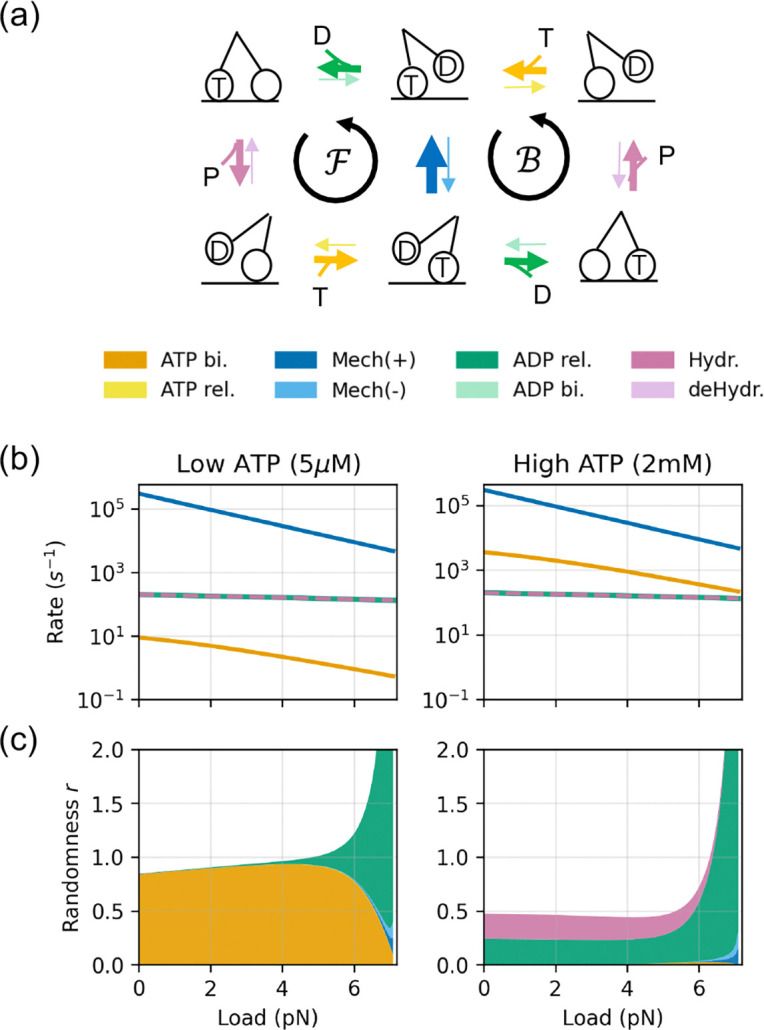
Dissecting Molecular Motor’s Randomness. (**a**) The 6-state model for kinesin [2]. (**b**) The bigger the load that the motor has to pull, the slower it runs, for given ATP energy sources [2]. (**c**) Decomposition of the motor’s randomness parameter r. The total randomness is decomposed into contributions from functional groups (aggregating forward F and backward B cycles), represented by the stacked areas. The upper boundary of the stacked areas are the randomness calculated by the kinesin model, fitted to Visscher et al. [29]. The motor becomes very inefficient when pulling heavy loads.

The power of Eq. (10) is illustrated by a new insight it gives into the molecular bases for the motor’s behavior. Fig. 2(b-c) reveals a shift in the stochasticity mechanism of kinesin: (i) For small loads (far from stalling), the motor is dominated by the *timing noise*, arising from the rate-limiting forward step(s)—the arrival of ATP at low [ATP] or the ADP release and ATP hydrolysis at high [ATP]. (ii) For big loads (near the stall force, green region), fluctuations are dominated by a *branching noise*. That is, near stall, the backward mechanical rate becomes comparable to the forward rate, and the ADP release step dominates the randomness because it controls the critical partitioning between the forward and backward cycles. This transition in fluctuation properties—between waiting for steps and committing to a step—is not obtainable from approaches that only analyze mean rates; it requires the fluctuation-response relations here.

### The CFT formalism enables scalable design.

To optimize the randomness parameter r by varying transition rate parameters $k_{ij}$, for example using zero-gradient conditions $\left(\nabla_{k}r=0\right)$ or by driving iterative gradient descent, the computational bottleneck entails evaluating the full gradient of r with respect to all rate constants. While analytical expressions for this exist, this evaluation becomes computationally prohibitive for large-scale models. Standard numerical approaches based on generating functions, such as finite differences combined with eigenvalue solvers (e.g., Koza’s method [24, 25]), are costly because they require recomputing the function for every parameter perturbation.

Here, we show that our $A^{-1}$ formalism breaks this bottleneck by ensuring algebraic closure: the gradient of the inverse matrix is fully determined by the inverse itself $\left(\partial A^{-1}=-A^{-1}(\partial A)A^{-1}\right)$. This allows evaluating both the gradients of mean and variance with a single inverse matrix, significantly improving scalability for locally connected networks—those with $N_{\text{edge}}=O\left(N_{\text{node}}\right)$, typical in biophysical models.

**Figure 3.**
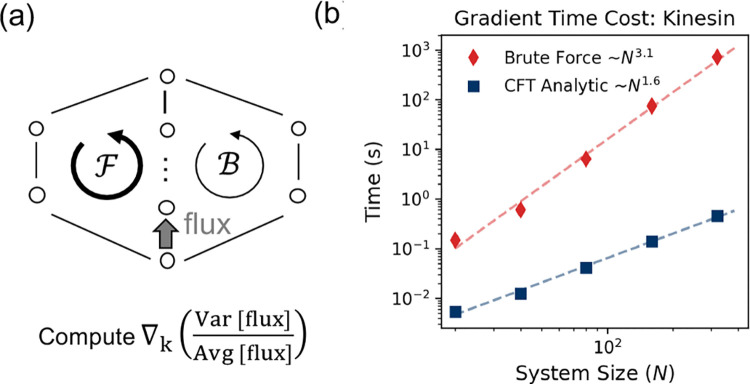
Breaking the Computational Bottleneck. **(a)** The Θ-shape topology used for benchmarking, representing a generalized motor model with multiple mechanical substeps to mimic the diffusive stepping process. **(b)** Scaling of the computation time for the gradient of the randomness parameter (ratio of variance and average of the flux) as a function of the number of mechanical substeps (and thus the system size).

Again for illustration, we use the kinesin model. To capture the continuous diffusive nature of the mechanical swing, we extend the single-hop transition in Liepelt and Lipowsky’s model [2] into a biased random walk across N substeps on a lattice (Fig. 3a). We take the chemical rates directly from their original model [27]. We benchmarked both methods by computing the full gradient under different numbers of mechanical substeps. As shown in Fig. 3(b), the brute-force method (red) scales as $O\left(N^{3.1}\right)$. In contrast, our $A^{-1}$ approach (blue) scales better, as $O\left(N^{1.6}\right)$, yielding a >100-fold speed-up for networks with $N_{\text{node}}\approx 100$. While our edge-centric algorithm theoretically scales less favorably for dense graphs $\left(N_{\text{edge}}=O\left(N_{\text{node}}^{2}\right)\right)$, we show in the Supplemental Material [27] that it remains computationally superior even on fully connected networks with sizes up to $N_{\text{node}}\approx 100$.

### Universal symmetries and kinetic bounds.

The geometric structure of the Jacobian A dictates universal principles that govern how any steady-state observables respond to perturbations in any ergodic network flow. From the identity $A^{-1}A=I$, we derive three sets of local response symmetries, valid for arbitrary CFT observables $\langle x\rangle \in \left\{\pi_{n},\tau_{ij},J_{c}\right\}$. This unifies and generalizes existing response relations [17, 20] within a single principle-based framework. See [27] for derivations.

On each node n, we derive a **Node Escaping Symmetry:**

(11) $$\sum_{l(\neq m)}\frac{k_{ml}}{\pi_{m}}\frac{\partial \langle x\rangle }{\partial k_{ml}}-\sum_{j(\neq n)}\frac{k_{nj}}{\pi_{n}}\frac{\partial \langle x\rangle }{\partial k_{nj}}=\delta_{\langle x\rangle ,\pi_{n}}$$

where the right-hand side is a Kronecker delta, introducing an additional factor of 1 only if $\langle x\rangle =\pi_{n}$. Physically, this corresponds to perturbing the site energy $E_{n}$ in an Arrhenius-like rate law $\left(k_{nj}\propto e^{E_{n}}\right)$ [17], which tunes all outgoing rates simultaneously $\left(\partial_{E_{n}}\equiv \sum_{j(\neq n)}k_{nj}\partial_{k_{nj}}\right)$. Eq. (11) thus generalizes the response equalities derived by Owen *et al.* [17] from state probabilities to arbitrary CFT observables. Applied to traffic or cycle fluxes, it reveals that these “node energy” perturbations scale all network fluxes proportional to local occupancy ($\partial_{E_{n}}\tau_{ij}=\pi_{n}\tau_{ij}$ and $\partial_{E_{n}}J_{c}=\pi_{n}J_{c}$).

On each edge ij, we have an Edge Reciprocity:

(12) $$\frac{1}{\pi_{i}}\frac{\partial \langle x\rangle }{\partial k_{ij}}+\frac{1}{\pi_{j}}\frac{\partial \langle x\rangle }{\partial k_{ji}}=2\delta_{\langle x\rangle ,\tau_{ij}}.$$

Physically, this symmetry governs the barrier height $B_{ij}$ perturbation in an Arrhenius-like rate law $\left(k_{ij},k_{ji}\propto e^{-B_{ij}}\right)$ [17], which tunes forward and backward rates symmetrically $\left(\partial_{B_{ij}}\equiv -k_{ij}\partial_{k_{ij}}-k_{ji}\partial_{k_{ji}}\right)$. Eq. (12) reveals that the response for $\langle x\rangle \neq \tau_{ij}$ is strictly proportional to the net flux on the perturbed edge $J_{ij}:=\pi_{i}k_{ij}-\pi_{j}k_{ji}:\partial_{B_{ij}}\langle x\rangle =-\left(J_{ij}/\pi_{i}\right)\partial_{k_{ij}}\langle x\rangle$. This implies that barrier perturbations cannot tune distributions $\pi_{n}$, cycle fluxes $J_{c}$, or other traffic terms $\tau_{uv}\;(uv\neq ij)$ if the edge is “locally” at equilibrium $\left(J_{ij}=0\right)$. They acquire control only through a nonzero nonequilibrium net flux $J_{ij}$. Together with our covariance decomposition in Eq. (9), these symmetries underpin the flux fluctuation-response identities derived by Aslyamov *et al.* [20], while extending their validity to arbitrary CFT observables.

On each cycle c, we find a **Cycle Symmetry:**

(13) $$\sum_{ij\in c^{+}}\frac{1}{\pi_{i}}\frac{\partial \langle x\rangle }{\partial k_{ij}}-\sum_{ji\in c^{-}}\frac{1}{\pi_{j}}\frac{\partial \langle x\rangle }{\partial k_{ji}}=2\delta_{\langle x\rangle ,J_{c}}.$$

This enforces a topological constraint: the cumulative sensitivity along any forward cycle must precisely balance that of its reversal (for observables $\langle x\rangle \neq J_{c}$, including all $\pi_{n}$, $\tau_{ij}$, and the other cycle fluxes $J_{c^{\prime }}$).

Our framework also provides the physical origins of established response bounds. By identifying the algebraic coefficients ${\text{Δ}}_{ij}$ and $\nabla_{ij}$ defined in Aslyamov and Esposito [18] as specific rate sensitivities [27]:

(14) $$\pi_{i}\left(1-{\text{Δ}}_{ij}\right)=\frac{\partial J_{ij}}{\partial k_{ij}}\;\text{and}\;\pi_{i}\left(1+\nabla_{ij}\right)=\frac{\partial \tau_{ij}}{\partial k_{ij}},$$

we elevate their algebraic constraints ($0\leq {\text{Δ}}_{ij}\leq 1$ and $\left|\nabla_{ij}\right|\leq {\text{Δ}}_{ij}$) into a physical kinetic hierarchy for the one-way fluxes $p_{ij}=\pi_{i}k_{ij}$:

(15) $$\pi_{i}\geq \frac{\partial p_{ij}}{\partial k_{ij}}\geq \frac{\partial p_{ji}}{\partial k_{ij}}\geq 0.$$

**Figure 4.**
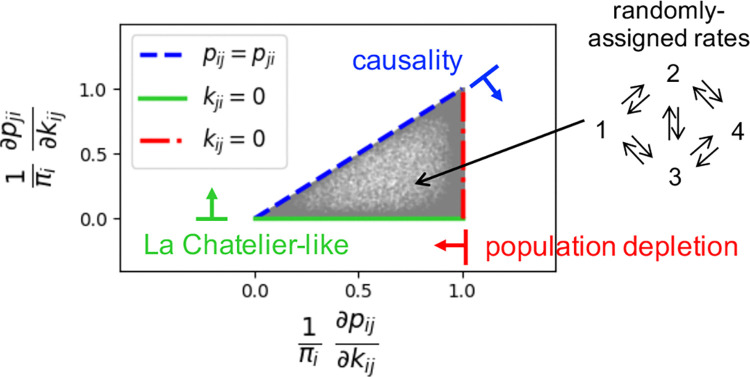
Universal Kinetic Bounds. The scatter plot numerically validates the derived sensitivity hierarchy using an ensemble of random 4-state networks as an example (schematic, upper right). Each grey dot represents the normalized response of the forward flux $p_{ij}$ (x-axis) versus the induced response of the reverse flux $p_{ji}$ (y-axis) to a perturbation in the forward rate $k_{ij}$. The feasible response region is strictly bounded by three physical limits: Population Depletion, Causality, and Le Chatelier-like Compensation.

As visualized in the triangular domain of Fig. 4, this hierarchy reflects the physical mechanisms governing nonequilibrium flow response, valid for any steady-state network flows.

The upper bound, $\pi_{i}\geq \partial_{k_{ij}}p_{ij}=\pi_{i}+k_{ij}\;\partial_{k_{ij}}\;\pi_{i}$, reflects *population depletion:* since increasing the escape rate $k_{ij}$ drains the source population $\left(\partial_{k_{ij}}\pi_{i}\leq 0\right)$, the flux cannot grow faster than the population size $\pi_{i}$. This limit is saturated as $k_{ij}\to 0$, where the perturbation is too weak to shift the steady-state population. The middle inequality, $\partial_{k_{ij}}p_{ij}\geq \partial_{k_{ij}}p_{ji}$, reflects *causality* $\left(\partial_{k_{ij}}J_{ij}\geq 0\right)$, showing a stronger push always increases net flux in the driven direction. This bound tightens to equality $\left(p_{ij}=p_{ji}\right)$ at zero-flux edges, characteristic of detailed balance or a topological bridge. Finally, the non-negativity of the induced reversed response $\left(\partial_{k_{ij}}p_{ji}\geq 0\right)$ captures a *Le Chatelier-like compensation*: pushing “particles” into the target state j increases its occupancy, which in turn drives an increased reverse backflow even when the reverse rate $k_{ji}$ remains fixed.

## Conclusions:

We have presented a force-based framework for nonequilibrium fluctuations and responses. It bridges between formal variational theory and computational utility. These relations reflect a deeper observable-force conjugacy, encoded in the inverse of a single Jacobian matrix. We demonstrated its dual utility: Analytically, it reveals principles of design and optimization, as shown in the kinesin molecular motor. Computationally, it scales efficiently for large networks. And it reveals general response relations and universal constraints on stochastic flows.

## Supplementary Material

Supplement 1

## Code Availability:

The codes used to produce the Figures are available at https://doi.org/10.5281/zenodo.18611573.
