# Supplementary material for: Fluctuation-Response Design Rules for Nonequilibrium Flows
Source: ArXiv. 2026 Feb 27:arXiv:2602.10957v2. Originally published 2026 Feb 11. Preprint. [Version 2] (PMC12919225)
Supplement: Supplement 1 [file NIHPP2602.10957v2-supplement-1.pdf]

Supplemental Material for:
"Fluctuation-Response Design Rules for Nonequilibrium Flows"

Ying-Jen Yang[1,*] and Ken A. Dill[1,2,3]

[1] *Laufer Center of Physical and Quantitative Biology, Stony Brook University*
[2] *Department of Physics, Stony Brook University*
[3] *Department of Chemistry, Stony Brook University*

[*] ying-jen.yang@stonybrook.edu

(Dated: March 2, 2026)

CONTENTS

Acknowledgments 5

References 5

I. The Caliber Force Framework and the Sparse Jacobian $\mathbf{A}$ 2
A. Stochastic Path Entropy of Markov Jump Processes 2
B. Fundamental Observables and the Asymptotic Constraints from Conservation 2
C. Conjugated Forces from Caliber Force Theory 3
D. Generic Construction of the Jacobian matrix $\mathbf{A}$ 4

II. The Response-Inverse-Matrix (RIM) relation 5
A. The General Score Function Derivation 5
B. The Alternative Derivation from Caliber Force Theory (CFT) 6
C. Derivation of the Covariance Decomposition 7

III. Details of Result 1: Covariance Decomposition of Kinesin 8
A. Model Topology and Transition Rates 8
B. Physical Assumptions 9
C. Parameter Sets 10
D. The Randomness Parameter as the Fano factor of the mechanical flux 10
E. Covariance Decomposition Implementation 10

IV. Details of Result 2: Scalable Gradient Evaluation 11
A. Exact Gradient via Algebraic Closure 11
B. Computational Complexity Analysis 12
1. Standard Spectral Method (Koza) 12
2. Algebraic RIM Method 13
3. Scaling Comparison 13
C. Numerical Benchmark: Extended Kinesin Model 13
1. Constructing the Extended Model 14
2. Scaling Results 14
D. Numerical Benchmark: Dense All-to-All Networks 14
1. Scaling Results 15
2. The Crossover Argument 15

V. Details of Result 3: Unifying Fluctuation-Response Relations 16
A. Derivation of the Three Sets of Local Response Relations 16
1. Class I: Edge Reciprocity 16
2. Class II: Node Escaping Symmetry 17
3. Class III: Cycle Symmetry 17
B. Generalizing Owen *et al.* [17] via Node Escaping Symmetry 17
Step 1: The Node Energy Shorthand 17

Step 2: Applying the Node Escaping Symmetry 17
Step 3: Determining the Constant via Homogeneity 18
Step 4: Final Result 18
C. How Traffic and Cycle Flux response to escaping rate tuning. 19
Step 1: The Symmetry Condition 19
Step 2: Determining the Constant via Homogeneity 19
Step 3: The Scaling Law 19
D. Barrier Tuning and the Net Flux Constraint 20
E. Generalizing the Flux Covariance from Aslyamov *et al.* [20] 20
1. Barrier Sensitivity (∂B_{ij}) 20
2. Force Sensitivity (∂F_{ij}) 21
Summary: Unifying the Decompositions 22
F. Translating the Kinetic Bounds from Aslyamov and Esposito [18] 22
Step 1: Expressing Δ_{ij}. 22
Step 2: Expressing ∇_{ij}. 22
Step 3: Deriving the Hierarchy. 23

I. THE CALIBER FORCE FRAMEWORK AND THE SPARSE JACOBIAN A

A. Stochastic Path Entropy of Markov Jump Processes

The path probability of Markov Jump processes with transition rates k_{ij} (from i to j) has an explicit expression. For path ω_t with time length t, it is

$$\mathcal{P}_{\mathbf{k}}(\omega_t) = p_0(i_0)[e^{k_{i_0 i_0}(t_1-0)}k_{i_0 i_1}]\cdots[e^{k_{i_{n-1}i_{n-1}}(t_n-t_{n-1})}k_{i_{n-1}i_n}]e^{k_{i_n i_n}(t-t_n)} \tag{S1}$$

where p_0 is the initial distribution, t_n is the n-th jump times, and $k_{ii} = -\sum_{j(\neq i)} k_{ij}$ is the negative of the escape rate at state i. By using counting variables—$N_{ij}(\omega_t)$ as the number of $i \mapsto j$ jumps and T_i as the total dwell time in state i—the path probability has a simpler form:

$$\mathcal{P}_{\mathbf{k}}(\omega_t) = p_0(i_0)\exp\left(\sum_{i\neq j} N_{ij}[\omega_t]\ln k_{ij} - \sum_i T_i[\omega_t]\sum_{j(\neq i)} k_{ij}\right). \tag{S2}$$

The *stochastic relative entropy* between the process with rates k_{ij} and a reference process with unit rates $u_{ij} = 1$ (both with the same initial distribution) is then

$$\mathcal{R}[\omega_t;\mathbf{k}] = \ln\frac{\mathcal{P}_{\mathbf{k}}(\omega_t)}{\mathcal{P}_{\mathbf{u}}(\omega_t)} = \sum_{i\neq j} N_{ij}(\omega_t)\ln k_{ij} - \sum_i T_i(\omega_t)\,\varepsilon_i(\mathbf{k}). \tag{S3}$$

where $\varepsilon_i = \sum_{j(\neq i)} k_{ij} - n_i$ is the escape rate offset by the out-degree of state i. This expression holds for arbitrary t. In mathematics, the path probability ratio (exponential of stochastic entropy) is also known as the *Radon-Nikodym derivatives*, typically denoted as $\mathrm{d}\mathbb{P}_{\mathbf{k}}/\mathrm{d}\mathbb{P}_{\mathbf{u}}$ in measure-theoretic probability theory. It captures all statistical differences between two processes ($\mathbf{k}$ and $\mathbf{u}$) as the expected value of any path observables $f(\omega_t)$ can be connected by

$$\langle f\rangle_{\mathbf{k}} = \sum_{\omega_t}\mathcal{P}_{\mathbf{k}}(\omega_t)f(\omega_t) = \sum_{\omega_t}\mathcal{P}_{\mathbf{u}}(\omega_t)\cdot\frac{\mathcal{P}_{\mathbf{k}}(\omega_t)}{\mathcal{P}_{\mathbf{u}}(\omega_t)}f(\omega_t) = \langle e^{\mathcal{R}}f\rangle_{\mathbf{u}}. \tag{S4}$$

Identifying time irreversibility (entropy production) as a type of stochastic relative entropy (with specific choices of reversals as the reference process) leads to their various fluctuation theorems (for a recent review, see [32]).

B. Fundamental Observables and the Asymptotic Constraints from Conservation

The counting variables (N_{ij}, T_i) are elemental as any time-extensive first-order path observables can be expressed by them:

$$\mathcal{B}(\omega_t) = \int_0^t f(i_t)\,\mathrm{d}t + \sum_n g(i_{t_{n-1}}, i_{t_n}) = \sum_i T_i\, f(i) + \sum_{i\neq j} N_{ij}\, g(i,j) \tag{S5}$$

where i_t is the state at time t and n sums over all jumps. However, they are degenerate due to the conservation of probability. We always have normalization constraints, $\sum_i T_i = t$, and in, the long-term, the transition counts must satisfy inward/outward flux balance (Kirchhoff's current law) [33]: $\sum_{j(\neq i)} N_{ij} = \sum_{j(\neq i)} N_{ji} + o(t)$ where $o(t)$ represents sublinear term $\lim_{t\to\infty} o(t)/t = 0$, which can also be denoted as $\sum_{j(\neq i)} N_{ij} \sim \sum_{j(\neq i)} N_{ji}$ by using standard asymptotic analysis notation $a \sim b \Leftrightarrow \lim_{t\to\infty} a/b = 1$.

The flux balance indicates that net fluxes $N_{ij} - N_{ji}$ are cyclic in the long term. Thus, to handle the constraints, we follow Caliber Force Theory (CFT) [26] and use the counting variables $\mathbf{X} = (\Phi_{ij}, T_n, \Psi_c)$. Traffic $\Phi_{ij} = N_{ij} + N_{ji}$ is the total flux, orthogonal to the net fluxes; the dwell time T_n excludes an arbitrary chosen reference node m in the network to handle normalization; $\Psi_c = N_{ab} - N_{ba}$ is the fundamental cycle flux where ab is the defining (directed) chord of the fundamental cycle c (obtained by adding one chord to an arbitrarily-chosen spanning tree of the state space network). The net flux on a given edge is simply the sum of the fundamental cycle fluxes that circulate through the edge:

$$N_{ij} - N_{ji} \sim \sum_c \Theta^c_{ij} \Psi_c \tag{S6}$$

where Θ^c_{ij} is the incidence matrix—it equals to 1 if edge ij aligns with the directed cycle c, -1 if the reverse ji aligns with the cycle c, and 0 if ij doesn't belong to cycle c. The set of fundamental counting observables $\mathbf{X} = (\Phi_{ij}, T_n, \Psi_c)$ thus spans (N_{ij}, T_i). This exploits the identity $N_{ij} = \frac{1}{2}[(N_{ij} + N_{ji}) + (N_{ij} - N_{ji})]$, which decomposes any count into a symmetric traffic part and an antisymmetric net flux part:

$$N_{ij} \sim \frac{\Phi_{ij} + \sum_c \Theta^c_{ij} \Psi_c}{2}, \; T_i = \begin{cases} T_n & \text{, if } i \neq m \\ t - \sum_{n(\neq m)} T_n & \text{, if } i = m \end{cases}. \tag{S7}$$

and thus all time extensive observables $\mathcal{B}(\omega_t)$.

C. Conjugated Forces from Caliber Force Theory

The stochastic path entropy $\mathcal{R}[\omega_t; \mathbf{k}]$ is an example of time-extensive observable $\mathcal{B}(\omega_t)$. We can thus rewrite it in terms of the fundamental counting variables $\mathbf{X} = (\Phi_{ij}, T_n, \Psi_c)$. Plugging Eq. (S7) into Eq. (S3) leads to

$$\mathcal{R}[\omega_t; \mathbf{k}] \sim \sum_{i\neq j} \frac{\Phi_{ij} + \sum_c \Theta^c_{ij} \Psi_c}{2} \ln k_{ij} - \sum_n T_n(\omega_t)\, \varepsilon_n - T_m(\omega_t)\, \varepsilon_m \tag{S8}$$

$$= \sum_{i\neq j} \frac{\Phi_{ij}}{2} \ln k_{ij} + \sum_{i\neq j} \frac{\sum_c \Theta^c_{ij} \Psi_c}{2} \ln k_{ij} - \sum_n T_n(\omega_t)\, \varepsilon_n - \left(t - \sum_{n(\neq m)} T_n(\omega_t) \right) \varepsilon_m. \tag{S9}$$

Using the symmetry of $\Phi_{ij} = \Phi_{ji}$ and exchanging the two summation of the second term, we get the asymptotic work-like form

$$\mathcal{R}[\omega_t; \mathbf{k}] \sim \sum_{i<j} \Phi_{ij}\, \mathfrak{F}_{\text{edge},ij} + \sum_c \Psi_c\, \mathfrak{F}_{\text{cycle},c} + \sum_n T_n\, \mathfrak{F}_{\text{node},n} - t\, \mathfrak{c} \tag{S10}$$

$$= \mathbf{X}(\omega_t) \cdot \mathfrak{F}(\mathbf{k}) - t\, \mathfrak{c}(\mathbf{k}). \tag{S11}$$

Here, the forces $\mathfrak{F}(\mathbf{k})$ are

$$\mathfrak{F}_{\text{edge},ij} = \frac{1}{2} \ln k_{ij} k_{ji}, \tag{S12}$$

$$\mathfrak{F}_{\text{node},n} = \sum_{i(\neq m)} (k_{mi} - 1) - \sum_{j(\neq n)} (k_{nj} - 1), \tag{S13}$$

$$\mathfrak{F}_{\text{cycle},c} = \frac{1}{2} \ln \frac{k_{i_0 i_1} k_{i_1 i_2} \cdots k_{i_\sigma i_0}}{k_{i_0 i_\sigma} k_{i_\sigma i_{\sigma-1}} \cdots k_{i_1 i_0}} \tag{S14}$$

where $i_0 i_1 ... i_\sigma i_0$ is the state sequence of the cycle c. The caliber (rate) $\mathfrak{c}(\mathbf{k}) = \varepsilon_m = \sum_{j(\neq m)} k_{im} - n_m$ has the value of the escape rate of the reference node m.

Eq. (S11) can be understood as follows. The stochastic path entropy $\mathcal{R}$ on the left quantifies the difference between the current process with rate $\mathbf{k}$ and a reference process with unit rate. The forces on the right are affinities driving edge exchange, node escaping, and cycle completion, with the counting variables $\mathbf{x}$ giving the statistical occurrence weights on the forces. The caliber term $(\mathfrak{c}\ t)$ is a "renormalization" factor. In fact, it has be shown in CFT [26] that the caliber is the log partition function for dynamics:

$$\begin{aligned}\lim_{t\to\infty}\frac{1}{t}\ln\sum_{\omega_t}\mathcal{P}_{\mathbf{u}}(\omega_t)e^{\mathfrak{F}\cdot\mathbf{X}(\omega_t)} &= \lim_{t\to\infty}\frac{1}{t}\ln\sum_{\omega_t}\mathcal{P}_{\mathbf{k}}(\omega_t)e^{-\mathcal{R}}e^{\mathfrak{F}\cdot\mathbf{X}(\omega_t)}\\ &= \lim_{t\to\infty}\frac{1}{t}\ln e^{t\mathfrak{c}}\sum_{\omega_t}\mathcal{P}_{\mathbf{k}}(\omega_t)\\ &= \mathfrak{c}(\mathfrak{F}).\end{aligned} \tag{S15}$$

Furthermore, its Legendre transform gives the average path entropy rate [26]:

$$-\mathfrak{s}_{\text{path}} = \lim_{t\to\infty}\frac{\mathcal{R}}{t} = \mathfrak{F}\cdot\langle\mathbf{x}\rangle - \mathfrak{c} \tag{S16}$$

where $\mathbf{x} = \mathbf{X}/t$ is the vector of the observable rates and $\langle\mathbf{x}\rangle$ is the vector of their steady-state averages, and the forces emerge naturally as the thermodynamics-like conjugate variables:

$$\mathfrak{F} = -\frac{\partial\mathfrak{s}_{\text{path}}}{\partial\langle\mathbf{x}\rangle}. \tag{S17}$$

Eq. (S11) shown in this paper is, in fact, the stochastic version of Eq. (S16) derived in [26], based on the law of large number in the long term limit $t\to\infty$:

$$\frac{\mathcal{R}}{t}\to(-\mathfrak{s}_{\text{path}})\ \text{and}\ \frac{\mathbf{X}}{t}\to\langle\mathbf{x}\rangle. \tag{S18}$$

D. Generic Construction of the Jacobian matrix A

We now consider how the stochastic path entropy $\mathcal{R}$ (Eqs. S3 and S11) varies when changing the process $\mathbf{k}$ of interest, under a fixed ω_t (and t):

$$\frac{\partial\mathcal{R}}{\partial\ln k_{ij}} = (N_{ij} - T_i k_{ij}) \tag{S19}$$

$$\sim \mathbf{x}\cdot\frac{\partial\mathfrak{F}}{\partial\ln k_{ij}} - t\frac{\partial\mathfrak{c}}{\partial\ln k_{ij}}. \tag{S20}$$

Thus, denoting $\lambda_{ij} = (N_{ij} - T_i k_{ij})/t$ and $\mathbf{x} = \mathbf{X}/t$, we arrive the key linear relationship between these Poissonian fluctuation on each transition ij and the CFT observable (rate) $\mathbf{x}$:

$$\boldsymbol{\lambda}(\omega_t) \sim \mathbf{A}(\mathbf{k})\ \mathbf{x}(\omega_t) - \nabla_{\ln\mathbf{k}}\mathfrak{c}(\mathbf{k}) \tag{S21}$$

where $\mathbf{A}_{(ij),\beta} = \partial\mathfrak{F}_\beta/\partial\ln k_{ij}$. One could also derive this linear map directly from how $\mathbf{X} = (\Phi_{ij}, T_n, \Psi_c)$ spans (N_{ij}, T_i) with Eq. (S7), but that derivation obscures the physical origin of $\mathbf{A}$'s sparsity. In our framework, the sparsity of $\mathbf{A}$ arises explicitly from the locality of the CFT forces $\mathfrak{F}$ (Eqs. S14), where each force depends only on a small, local subset of transition rates.

By defining $\lambda'_{mj} = \lambda_{mj} + (\partial\mathfrak{c}/\partial\ln k_{mj}) = \lambda_{mj} + k_{mj}$ and denoting $\mathbf{x} = \mathbf{X}/t = (\Phi_{ij}/t, T_n/t, \Psi_c/t) = (\phi_{ij}, f_n, \psi_c)$, we can rewrite Eq. (S21) into a simpler linear map:

$$\tilde{\boldsymbol{\lambda}} \sim \mathbf{A}\mathbf{x} \tag{S22}$$

where $\tilde{\boldsymbol{\lambda}}$ has components λ_{ij} for $i(\neq m)$ and λ'_{ij} for $i = m$. By ordering the edge properly, the sparse matrix $\mathbf{A}$ has a generic form as shown in Fig. S1 (an example is given in the main text):

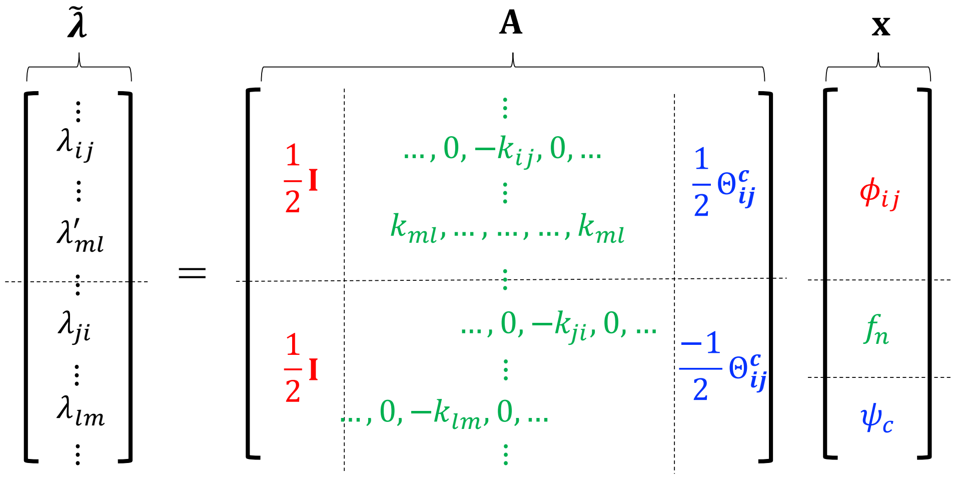

Figure S1. The matrix maps the CFT observable vector $\mathbf{x} = (\phi, f, \psi)^\mathsf{T}$ to the vector of independent noise sources $\tilde{\boldsymbol{\lambda}}$. The sparse geometry consists of three distinct vertical blocks: (Left, Red) a diagonal identity block scaling edge traffic; (Middle, Green) a sparse block containing transition rates linking node frequencies to noise; and (Right, Blue) a topological block containing the cycle incidence matrix Θ^c_{ij}. The vector $\tilde{\boldsymbol{\lambda}}$ accounts for the shifted noise λ'_{ml} for transitions originating from the reference state m.

II. THE RESPONSE-INVERSE-MATRIX (RIM) RELATION

We here derive one of our main result:

$$\frac{\partial\langle x_\alpha\rangle}{\partial \ln k_{ij}} = t\,\mathrm{Cov}[x_\alpha, \lambda_{ij}] \sim \pi_i k_{ij}\,[\mathbf{A}^{-1}]_{\alpha,(ij)} = \pi_i k_{ij}\,\nabla_{\mathfrak{F}_\alpha} \ln k_{ij}. \tag{S23}$$

The first equality is known and recently discussed by Zheng and Lu [19]. We show here that, for CFT observables $\mathbf{x}$, all these responses and covariances are encoded in the inverse of a sparse Jacobian $\mathbf{A}$.

A. The General Score Function Derivation

Consider a generic path observable $f(\omega_t)$ whose functional form is independent to k_{ij}. It is straightforward to show that the average susceptibility to $\ln k_{ij}$ perturbation (i.e. $\partial_{\ln k} \equiv k\partial_k$) is the covariance between f and the independent noise sources λ_{ij}:

$$k_{ij}\frac{\partial}{\partial k_{ij}}\sum_{\omega_t}\mathcal{P}_\mathbf{k}(\omega_t)f(\omega_t) = \sum_{\omega_t}\frac{\partial \mathcal{P}_\mathbf{k}}{\partial \ln k_{ij}}\,f(\omega_t) = \sum_{\omega_t}\mathcal{P}_\mathbf{k}(\omega_t)\,\frac{\partial \ln \mathcal{P}_\mathbf{k}}{\partial \ln k_{ij}}\,f(\omega_t) \tag{S24}$$

$$= \sum_{\omega_t}\mathcal{P}_\mathbf{k}(\omega_t)\,\frac{\partial \mathcal{R}}{\partial \ln k_{ij}}\,f(\omega_t) = t\sum_{\omega_t}\mathcal{P}_\mathbf{k}(\omega_t)\,\lambda_{ij}\,f(\omega_t) = t\,\mathrm{Cov}(\lambda_{ij}, f) \tag{S25}$$

where we have used Eq. (S19) and that λ_{ij} is zero mean. Applying it to CFT observables $\mathbf{x}$ and taking the long-term limit $t \to \infty$ leads to the relation between steady-state average responses and asymptotic covariances:

$$\frac{\partial\langle x_\alpha\rangle}{\partial \ln k_{ij}} = \lim_{t\to\infty} t\,\mathrm{Cov}(\lambda_{ij}, x_\alpha) \tag{S26}$$

where x_α can be any of (ϕ_{ij}, f_n, ψ_c).

To link these to the Jacobian $\mathbf{A} = \nabla_{\ln \mathbf{k}}\mathfrak{F}$, we use the (asymptotic) linear relation

$$\boldsymbol{\lambda} - \nabla_{\ln \mathbf{k}}\mathfrak{c} \sim \mathbf{A}\mathbf{x} \Rightarrow \mathbf{x} \sim \mathbf{A}^{-1}(\boldsymbol{\lambda} - \nabla_{\ln \mathbf{k}}\mathfrak{c}). \tag{S27}$$

The inverse matrix $\mathbf{A}^{-1}$ exists because CFT forces constitute a coordinate for the parameter space [26], i.e. $\mathbf{k} \leftrightarrow \mathfrak{F}$ is one-to-one. Plugging Eq. (S27) into Eq. (S26) leads to

$$\frac{\partial\langle x_\alpha\rangle}{\partial \ln k_{ij}} = \lim_{t\to\infty} t\,\mathrm{Cov}(\lambda_{ij}, [\mathbf{A}^{-1}(\boldsymbol{\lambda} - \nabla_{\ln \mathbf{k}}\mathfrak{c})]_\alpha). \tag{S28}$$

Now, we evaluate the covariance of the noise sources $\lambda_{ij} = (N_{ij} - T_i k_{ij})/t$. Since elementary jumps follow Poisson statistics in the short-time limit and remain independent across different edges, the variance of the count N_{ij} scales with its mean $t\pi_i k_{ij}$. This yields the diagonal covariance structure [19]:

$$\lim_{t\to\infty} t \cdot \mathrm{Cov}[\lambda_{ij}, \lambda_{ab}] = \langle N_{ij}/t\rangle \delta_{i,a}\delta_{j,b} = \pi_i k_{ij}\, \delta_{i,a}\, \delta_{j,b}. \tag{S29}$$

This reduces Eq. (S28) into

$$\frac{\partial\langle x_\alpha\rangle}{\partial \ln k_{ij}} = \pi_i k_{ij} [\mathbf{A}^{-1}]_{\alpha,(ij)}. \tag{S30}$$

B. The Alternative Derivation from Caliber Force Theory (CFT)

Here, we provide an alternative derivation from CFT, demonstrating the consistency of RIM with CFT's fluctuation-response equalities [26]. Given that the caliber $\mathfrak{c}(\mathfrak{F})$ functions as the log partition function (as revisited in Eq. S15), its first derivatives yield steady-state averages:

$$\langle x_\alpha\rangle = \frac{\partial \mathfrak{c}}{\partial \mathfrak{F}_\alpha}, \tag{S31}$$

and its second derivatives yield covariances:

$$\frac{\partial\langle x_\alpha\rangle}{\partial \mathfrak{F}_\beta} = \frac{\partial\langle x_\beta\rangle}{\partial \mathfrak{F}_\alpha} = \frac{\partial^2 \mathfrak{c}}{\partial \mathfrak{F}_\alpha \partial \mathfrak{F}_\beta} = \lim_{t\to\infty} t \cdot \mathrm{Cov}(x_\alpha, x_\beta) \tag{S32}$$

where (x_α, x_β) can be any pair from (f_n, ϕ_{ij}, ψ_c), and $\mathfrak{F}_\alpha$ is the conjugate force of x_α.

Consider a rate perturbation of an arbitrary mean observable, $\partial\langle x\rangle/\partial k_{ij}$. When $i \neq m$, this perturbation affects the node force $\mathfrak{F}_i$, the edge force $\mathfrak{F}_{ij}$, and the cycle forces $\mathfrak{F}_c$ for all cycles c passing through edge ij. Therefore, in the force coordinate system, we can expand the rate responses in terms of CFT force responses:

$$\begin{aligned}\frac{\partial\langle x\rangle}{\partial k_{ij}} =& \frac{\partial\langle x\rangle}{\partial \mathfrak{F}_i}\frac{\partial \mathfrak{F}_i}{\partial k_{ij}} + \frac{\partial\langle x\rangle}{\partial \mathfrak{F}_{ij}}\frac{\partial \mathfrak{F}_{ij}}{\partial k_{ij}} + \sum_c \frac{\partial\langle x\rangle}{\partial \mathfrak{F}_c}\frac{\partial \mathfrak{F}_c}{\partial k_{ij}} \\ =& \frac{\partial\langle x\rangle}{\partial \mathfrak{F}_i}(-1) + \frac{\partial\langle x\rangle}{\partial \mathfrak{F}_{ij}}\left(\frac{1}{2k_{ij}}\right) + \sum_c \frac{\partial\langle x\rangle}{\partial \mathfrak{F}_c}\left(\frac{1}{2k_{ij}}\Theta_c^{ij}\right)\end{aligned} \tag{S33}$$

where Θ_c^{ij} is the (directed) incidence matrix: it equals 1 if ij aligns with c, -1 if ij anti-aligns with c, and 0 otherwise. Now, using the fluctuation-response relations in CFT:

$$\frac{\partial\langle x\rangle}{\partial \mathfrak{F}_i} = \lim_{t\to\infty} t \cdot \mathrm{Cov}(x, f_i) \tag{S34}$$

$$\frac{\partial\langle x\rangle}{\partial \mathfrak{F}_{ij}} = \lim_{t\to\infty} t \cdot \mathrm{Cov}(x, f_{ij} + f_{ji}) \tag{S35}$$

$$\frac{\partial\langle x\rangle}{\partial \mathfrak{F}_c} = \lim_{t\to\infty} t \cdot \mathrm{Cov}(x, f_{ab} - f_{ba}). \tag{S36}$$

However, since asymptotic flux balance implies $\lim_{t\to\infty}\sum_j (f_{ij} - f_{ji}) = 0$, the net edge flux $f_{ij} - f_{ji}$ is asymptotically spanned by the fundamental cycle fluxes:

$$\lim_{t\to\infty}(f_{ij} - f_{ji}) - \sum_c \Theta_c^{ij}(f_{ab} - f_{ba}) = 0. \tag{S37}$$

Substituting these into Eq. (S33), we obtain:

$$\begin{aligned}\frac{\partial\langle x\rangle}{\partial k_{ij}} &= \lim_{t\to\infty} t \cdot \left[-\mathrm{Cov}(x, f_i) + \frac{1}{2k_{ij}}\left(\mathrm{Cov}(x, f_{ij} + f_{ji}) + \sum_c \Theta_c^{ij}\mathrm{Cov}(x, f_{ab} - f_{ba})\right)\right] \\ &= \lim_{t\to\infty} t \cdot \left[-\mathrm{Cov}(x, f_i) + \frac{1}{2k_{ij}}\left(\mathrm{Cov}(x, f_{ij} + f_{ji}) + \mathrm{Cov}(x, f_{ij} - f_{ji})\right)\right] \\ &= \lim_{t\to\infty} t \cdot \left[-\mathrm{Cov}(x, f_i) + \frac{1}{k_{ij}}\mathrm{Cov}(x, f_{ij})\right].\end{aligned}$$

Thus, by identifying $\lambda_{ij} = f_{ij} - f_i k_{ij}$, we have shown (for $i \neq m$):

$$k_{ij}\frac{\partial\langle x\rangle}{\partial k_{ij}} = \lim_{t\to\infty} t\cdot \mathrm{Cov}[x, \lambda_{ij}].$$

When $i = m$, the perturbation k_{ij} affects all node forces (while the edge and cycle contributions remain identical to the $i \neq m$ case, denoted by "…"). We get:

$$\begin{aligned}
\frac{\partial\langle x\rangle}{\partial k_{ij}} &= \sum_{n(\neq i)} \frac{\partial\langle x\rangle}{\partial \mathfrak{F}_n} \underbrace{\frac{\partial \mathfrak{F}_n}{\partial k_{ij}}}_{=1} + \cdots \\
&= \lim_{t\to\infty} t \sum_{n(\neq i)} \mathrm{Cov}(x, f_n) + \cdots \\
&= \lim_{t\to\infty} t\cdot \mathrm{Cov}\left(x, \sum_{n(\neq i)} f_n\right) + \cdots \\
&= \lim_{t\to\infty} t\cdot \mathrm{Cov}(x, 1 - f_i) + \cdots \\
&= -\lim_{t\to\infty} t\cdot \mathrm{Cov}(x, f_i) + \cdots
\end{aligned} \tag{S38}$$

where we have used the linearity of covariance and the fact that a constant shift in the variable does not change the covariance. Eq. (S38) completes the CFT-based derivation for Eq. (12) of the main text.

C. Derivation of the Covariance Decomposition

First, we note that the covariance is invariant under constant shifts, i.e., $\mathrm{CoV}[X, Y + c] = \mathrm{CoV}[X, Y]$. This implies that the shifted noise sources at the reference node, $\lambda'_{mj} = \lambda_{mj} + k_{mj}$, contribute identically to the covariance as the raw noise λ_{mj}. We can thus treat all transitions uniformly in the covariance calculation.

The covariance expansion in terms of edge responses can be derived with a direct calculation using the RIM relation:

$$\begin{aligned}
\lim_{t\to\infty} t\cdot \mathrm{CoV}[x, x'] &= \lim_{t\to\infty} t\cdot \mathrm{CoV}[\sum_{ab} A^{-1}_{x,ab}\lambda_{ab}, \sum_{ij} A^{-1}_{x',ij}\lambda_{ij}] \\
&= \sum_{ab}\sum_{ij} \lim_{t\to\infty} t\cdot A^{-1}_{x,ab}A^{-1}_{x',ij}\mathrm{CoV}[\lambda_{ab}, \lambda_{ij}] \\
&= \sum_{ab}\sum_{ij} A^{-1}_{x,ab}A^{-1}_{x',ij}\,(\pi_i k_{ij}\delta_{ai}\delta_{bj}) \\
&= \sum_{ij} \pi_i k_{ij} A^{-1}_{x,ij} A^{-1}_{x',ij}.
\end{aligned}$$

This gives fast evaluation of covariances with $\mathbf{A}^{-1}$.

To explicitly recover the geometric interpretation (Result 1 in the main text), we introduce the **normalized noise source** $\hat{\lambda}_{ij}$, defined by rescaling λ_{ij} with its asymptotic standard deviation $\sigma_{ij} = \sqrt{\pi_i k_{ij}/t}$:

$$\hat{\lambda}_{ij} \equiv \frac{\lambda_{ij}}{\sqrt{\pi_i k_{ij}/t}}. \tag{S39}$$

These normalized sources are orthonormal in the long term, satisfying $\lim_{t\to\infty} t\cdot \mathrm{Cov}[\hat{\lambda}_{ij}, \hat{\lambda}_{ab}] = \delta_{ia}\delta_{jb}$. Consequently, the covariance contribution of a specific transition ij is simply the product of the projections of the observables onto this unit noise

vector (via RIM):

$$\lim_{t\to\infty} t \cdot \mathrm{Cov}[x, \hat{\lambda}_{ij}] = \lim_{t\to\infty} t \cdot \mathrm{Cov}[\sum_{ab} A^{-1}_{x,ab}\lambda_{ab}, \frac{\lambda_{ij}}{\sqrt{\pi_i k_{ij}/t}}]$$
$$= \frac{1}{\sqrt{\pi_i k_{ij}/t}}(\pi_i k_{ij} A^{-1}_{x,ij})$$
$$= \sqrt{t\pi_i k_{ij}}\, A^{-1}_{x,ij}. \tag{S40}$$

Substituting this back into the covariance sum recovers the **Parseval-like identity** (Eq. 11 in the main text):

$$\mathrm{Cov}[x, x'] \sim \sum_{ij} \mathrm{Cov}[x, \hat{\lambda}_{ij}] \cdot \mathrm{Cov}[x', \hat{\lambda}_{ij}]. \tag{S41}$$

This confirms that the total fluctuation is the sum of squared sensitivities projected onto independent microscopic noise sources.

III. DETAILS OF RESULT 1: COVARIANCE DECOMPOSITION OF KINESIN

To generate the covariance landscapes in Fig. 2 of the main text, we utilized the 6-state network model for Kinesin developed by Liepelt and Lipowsky [2]. This model captures the chemomechanical coupling of the motor under varying external loads (F) and ATP concentrations ([ATP]).

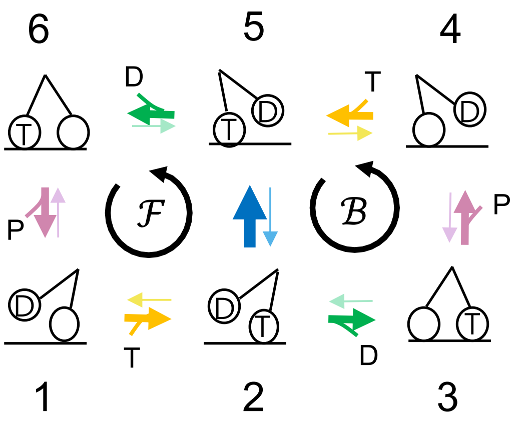

Figure S2. **Topology of the 6-state kinesin network.** The states are indexed $1-6$ based on the chemical composition of the trailing (left) and leading (right) heads (T = ATP, D = ADP). The network consists of a forward mechanical cycle $\mathcal{F}$ (left loop, physiological) and a backward hydrolysis cycle $\mathcal{B}$ (right loop). The central blue arrows denote the mechanical transitions ($2 \leftrightarrow 5$).

A. Model Topology and Transition Rates

The model network consists of 6 chemical states (Fig. S2). The transitions form two primary cycles: the forward mechanical stepping cycle ($\mathcal{F}$) and the backward hydrolysis cycle ($\mathcal{B}$). For consistency with the provided Python code, we note that the code uses 0-based indexing (states 0-5), which corresponds directly to states 1-6 in this text (e.g., Code state 0 is State 1).

The transition rate from state i to j is parameterized as a function of the external load F and chemical concentrations $[X]$:

$$k_{ij}(F, [X]) = k^0_{ij} \cdot \mathcal{I}_{ij}([X]) \cdot \Phi_{ij}(F). \tag{S42}$$

Here, k^0_{ij} is the zero-load rate constant. The concentration factor $\mathcal{I}_{ij}([X])$ is $[X]$ for binding transitions and 1 otherwise. The force-dependence factors $\Phi_{ij}(F)$ are modeled as follows:

Mechanical Transitions ($2 \leftrightarrow 5$): These steps involve the physical translocation of the motor ($d = 8$ nm) and follow a Bell-like exponential dependence:

$$\Phi_{25}(F) = \exp\left(-\theta \frac{Fd}{k_B T}\right), \quad \text{(Forward Step)} \tag{S43}$$

$$\Phi_{52}(F) = \exp\left((1-\theta) \frac{Fd}{k_B T}\right). \quad \text{(Backward Step)} \tag{S44}$$

where θ is the load distribution factor.

Chemical Transitions ($i \leftrightarrow j$): Chemical transitions are modeled with a sigmoid-like dependence to account for the mechanical strain on the catalytic domain. This introduces the **load sensitivity factor** χ_{ij}:

$$\Phi_{ij}(F) = \frac{2}{1 + \exp\left(\chi_{ij} \frac{Fd}{k_B T}\right)}. \tag{S45}$$

The parameter χ_{ij} characterizes the distance to the transition state in the chemical coordinate space under load.

B. Physical Assumptions

Based on the definitions above, the model from Liepelt and Lipowsky relies on the following physical assumptions which impose constraints on the parameters k^0_{ij} and χ_{ij}:

- **Head Symmetry and Thermodynamic Constraints:** Since the two motor heads are identical, the kinetics of chemical transitions are generally independent of the head's position. Consequently, the model assigns identical zero-load rates to corresponding steps in the forward and backward cycles:

$$\begin{aligned} k^0_{12} = k^0_{45} \quad &\text{(ATP Binding)}; \quad k^0_{56} = k^0_{23} \quad \text{(ADP Release)} \\ k^0_{65} = k^0_{32} \quad &\text{(ADP Rebinding)}; \quad k^0_{61} = k^0_{34} \quad \text{(Hydrolysis)} \\ k^0_{16} = k^0_{43} \quad &\text{(Synthesis)} \end{aligned} \tag{S46}$$

  The only exception is the ATP unbinding rate k^0_{54}. Unlike strict symmetry ($k^0_{54} = k^0_{21}$), this rate is rigorously determined by the loop closure condition to ensure thermodynamic consistency. Fundamentally, the cycle affinity $\mathcal{A}$—defined kinetically as the log-ratio of the product of forward rates to backward rates around a closed loop—must match the physical thermodynamic free energy difference ΔG that serves as the "battery" of the cycle (e.g., $\Delta\mu_{\text{ATP}}$). Since the mechanical step introduces a strong kinetic asymmetry ($k^0_{25} \neq k^0_{52}$), satisfying this potential condition across the coupled forward and backward cycles mandates a compensatory adjustment in the unbinding rate:

$$k^0_{54} = k^0_{21} \left(\frac{k^0_{52}}{k^0_{25}}\right)^2. \tag{S47}$$

  This ensures that the model respects detailed balance at equilibrium and correctly describes the dissipation away from equilibrium.

- **No Mechanical Substeps:** The mechanical step occurs in a single transition ($2 \leftrightarrow 5$) without intermediate substeps.

- **Symmetric Load Dependence for Chemical Transitions:** The load dependence of chemical transitions is assumed to be symmetric, meaning the distance to the transition state is equal for forward and backward rates. This implies: $\chi_{ij} = \chi_{ji}$. Consequently, $\Phi_{ij}(F) = \Phi_{ji}(F)$, meaning the external load scales the forward and reverse chemical rates by the same factor.

- **Identical Kinetics for ADP Release and Hydrolysis:** To reduce the number of free parameters, the model assumes that the ADP release steps ($5 \to 6, 2 \to 3$) and the Hydrolysis steps ($6 \to 1, 3 \to 4$) share identical kinetic properties. This implies equality in both their zero-load rates and their load sensitivities ($k^0_{56} = k^0_{61}$ and $\chi_{56} = \chi_{61}$). Consequently, these distinct chemical transitions proceed with identical rates under all load conditions:

$$k_{56}(F) = k_{61}(F) \quad \text{and} \quad k_{23}(F) = k_{34}(F). \tag{S48}$$

Parameter	Value	Description
θ	0.30	Load distribution factor
k_{25}^0	3.0×10^5	Mechanical fwd rate
k_{52}^0	0.24	Mechanical bwd rate
k_{12}^0	1.8	ATP binding rate
k_{56}^0	200.0	ADP release rate
k_{61}^0	200.0	Hydrolysis rate
χ_{12}	0.25	Load factor (ATP)
χ_{56}	0.05	Load factor (ADP/Hydr)

Table S1. Parameter set used for the Kinesin model (Visscher et al.). Rates are in units of s^{-1} (first order) or $\mu M^{-1}s^{-1}$ (second order). Only one rate is listed for symmetric pairs.

C. Parameter Sets

We utilized the parameter set Liepelt and Lipowsky [2] fitted to the optical trap data from Visscher *et al.* [29]. We selected this specific dataset because it provides comprehensive experimental measurements of the motor's **randomness parameter** (Fano factor—the variance normalized by the mean—of motor's velocity) under varying loads and ATP concentrations, which is essential for validating our fluctuation analysis. The explicit values used in our calculations are listed in Table S1.

D. The Randomness Parameter as the Fano factor of the mechanical flux

Denote the stochastic motor displacement at time t as $\Delta x(t) = d \cdot (N_+(t) - N_-(t))$, where $d = 8$ nm is the step size, and $N_+(t)$ and $N_-(t)$ denote the cumulative counts of forward ($2 \to 5$) and backward ($5 \to 2$) mechanical steps up to time t. We define the time-averaged net flux of the mechanical step as the random variable:

$$\psi \equiv \frac{N_+(t) - N_-(t)}{t}. \tag{S49}$$

By the Law of Large Numbers, the time-averaged quantities converge to their ensemble means as $t \to \infty$:

$$\lim_{t\to\infty} d \cdot \psi = \langle v \rangle = d \cdot \langle \psi \rangle, \tag{S50}$$

$$\lim_{t\to\infty} \frac{\text{Var}(\Delta x(t))}{t} = 2D = \lim_{t\to\infty} d^2 \cdot t \cdot \text{Var}(\psi). \tag{S51}$$

The randomness parameter r is defined as the Fano factor of the flux:

$$r = \frac{2D}{\langle v \rangle d} = \lim_{t\to\infty} \frac{t \cdot \text{Var}(\psi)}{\langle \psi \rangle}. \tag{S52}$$

E. Covariance Decomposition Implementation

To spatially resolve the sources of noise, we partitioned the 14 directed transitions of the network into 8 physically distinct groups, denoted here as the set $\mathcal{G}$. The total Fano factor r of the motor velocity is the sum of the randomness contributions S_g from each group:

$$r = \sum_{g\in\mathcal{G}} S_g. \tag{S53}$$

Based on the RIM relation (Eq. 11 in the main text), the contribution S_g is calculated by summing the squared-sensitivity projections of the individual edges (i, j) belonging to that group:

$$S_g = \frac{1}{\langle \psi \rangle} \sum_{(i,j)\in g} \frac{1}{\pi_i k_{ij}} \left(\frac{\partial \langle \psi \rangle}{\partial \ln k_{ij}} \right)^2 \sim \frac{1}{\langle \psi \rangle} \sum_{(i,j)\in g} \pi_i k_{ij} \left([\mathbf{A}^{-1}]_{\psi,(ij)} \right)^2. \tag{S54}$$

The groups are defined to distinguish between the forward reaction steps (driving the cycles) and the reverse reaction steps (opposing the cycles).

Crucially, we aggregate the chemical transitions from both the forward and backward cycles into single categories. This reflects the physical reality that corresponding chemical steps in both cycles are governed by the same environmental parameters (e.g., [ATP] or [ADP]), acting effectively as a single control knob. Since an experimental perturbation to the concentration affects these rates simultaneously, summing their contributions provides a more physically meaningful measure of sensitivity than treating them in isolation. This aggregation also simplifies the covariance landscape for clearer visualization. The eight groups are:

- **ATP Binding:** Aggregates the forward binding rates from both cycles ($0 \to 1, 3 \to 4$) vs. Release ($1 \to 0, 4 \to 3$).
- **Mechanical Step:** Forward ($1 \to 4$) vs. Backward ($4 \to 1$).
- **ADP Release:** Aggregates the forward release rates from both cycles ($4 \to 5, 1 \to 2$) vs. Binding ($5 \to 4, 2 \to 1$).
- **Hydrolysis:** Aggregates the forward hydrolysis rates from both cycles ($5 \to 0, 2 \to 3$) vs. Synthesis ($0 \to 5, 3 \to 2$).

This decomposition allows us to identify whether the total randomness r is dominated by the mechanical stepping itself or by the stochasticity of specific chemical steps.

IV. DETAILS OF RESULT 2: SCALABLE GRADIENT EVALUATION

In this section, we demonstrate how the algebraic closure of the inverse Jacobian matrix allows for computing the exact gradient of the randomness parameter with respect to *all* rate constants in a single calculation step, avoiding expensive numerical perturbations.

A. Exact Gradient via Algebraic Closure

To apply the RIM formalism, we explicitly select the mechanical transition ($2 \leftrightarrow 5$) as the defining chord for the fundamental mechanical cycle. Consequently, the flux observable ψ corresponds to a basis vector in the Jacobian $\mathbf{A}$. The covariance decomposition gives:

$$\lim_{t\to\infty} t \cdot \mathrm{Var}(\psi) = \sum_{(i,j)} \pi_i k_{ij} \left([\mathbf{A}^{-1}]_{\psi,(ij)}\right)^2 . \tag{S55}$$

Thus, the randomness parameter is simply:

$$r = \frac{1}{\langle\psi\rangle} \sum_{i\neq j} \pi_i k_{ij} \left([\mathbf{A}^{-1}]_{\psi,(ij)}\right)^2 . \tag{S56}$$

Our goal is to compute its gradient vector $\nabla_{\ln \mathbf{k}} r$.

The calculation is a straightforward calculus. The key step is differentiating the inverse matrix using the relation:

$$\partial_{k_{ij}} \mathbf{A}^{-1} = -\mathbf{A}^{-1} \left(\partial_{k_{ij}} \mathbf{A}\right) \mathbf{A}^{-1}. \tag{S57}$$

This is derived by differentiating the identity $\mathbf{I} = \mathbf{A}\mathbf{A}^{-1}$. Based on the structure of the Jacobian $\mathbf{A}$ (Fig. S1), where rows are indexed by edges (forces) and columns by observables (frequencies f and fluxes), the derivative matrix $\partial_{k_{ij}} \mathbf{A}$ is extremely sparse. Its non-zero components appear **only** in the columns corresponding to node frequencies.

We can thus reduce the derivative relation into the following explicit component forms:

1. **For a non-reference node ($i \neq m$):** The rate k_{ij} appears linearly with a coefficient of -1 at the intersection of row (ij) and column f_i. Thus, $\partial_{k_{ij}}\mathbf{A}$ has a single non-zero entry of -1:

$$\begin{aligned}\partial_{k_{ij}}\mathbf{A}^{-1}_{\alpha,ab} &= -\sum_{cd,\beta}\mathbf{A}^{-1}_{\alpha,cd}\,(\partial_{k_{ij}}\mathbf{A})_{cd,\beta}\,\mathbf{A}^{-1}_{\beta,ab},\\ &= -\sum_{cd,\beta}\mathbf{A}^{-1}_{\alpha,cd}\,(-\delta_{c,i}\delta_{d,j}\,\delta_{\beta,f_i})\,\mathbf{A}^{-1}_{\beta,ab},\\ &= +\mathbf{A}^{-1}_{\alpha,ij}\,\mathbf{A}^{-1}_{f_i,ab}.\end{aligned} \tag{S58}$$

2. **For the reference node ($i = m$):** Due to the normalization constraint $\sum f_n = 1$, the rate k_{mj} appears with a coefficient of $+1$ in every column corresponding to an independent frequency f_n. Thus, the derivative is a sum over all basis nodes n:

$$\begin{aligned}\partial_{k_{mj}}\mathbf{A}^{-1}_{\alpha,ab} &= -\sum_{cd,\beta}\mathbf{A}^{-1}_{\alpha,cd}\,(\partial_{k_{mj}}\mathbf{A})_{cd,\beta}\,\mathbf{A}^{-1}_{\beta,ab},\\ &= -\sum_{cd,\beta}\mathbf{A}^{-1}_{\alpha,cd}\,(\delta_{c,m}\delta_{d,j}\sum_n\delta_{\beta,f_n})\,\mathbf{A}^{-1}_{\beta,ab},\\ &= -\mathbf{A}^{-1}_{\alpha,mj}\,\sum_n\mathbf{A}^{-1}_{f_n,ab}.\end{aligned} \tag{S59}$$

Finally, since the derivatives of π_i (mean frequency $\langle f_i\rangle$) and $\langle\psi\rangle$ can also be evaluated directly using $\mathbf{A}^{-1}$ (via RIM), the full gradient vector ∇r is evaluated efficiently in a single pass once $\mathbf{A}^{-1}$ is computed.

B. Computational Complexity Analysis

Here, we compare the theoretical scaling of our algebraic approach against the standard spectral deformation technique (Koza's method [24]) for a general network with N nodes and M physical links. The number of rate parameters is $N_{\text{param}} = 2M$ (forward and backward rates).

To rigorously analyze the scaling crossover, we express the total computational time $\mathcal{T}$ as the sum of a one-time pre-calculation cost ($\mathcal{T}_{\text{setup}}$) and the cumulative cost of evaluating gradients for all parameters ($\mathcal{T}_{\text{iter}}$):

$$\mathcal{T}_{\text{total}} = \mathcal{T}_{\text{setup}} + N_{\text{param}}\cdot\mathcal{T}_{\text{step}}. \tag{S60}$$

1. *Standard Spectral Method (Koza)*

The standard approach calculates the cumulants of a current via the generating function. One constructs a parameter-dependent generator matrix (or tilted matrix) $\tilde{\mathbf{W}}(s)$ of size $N\times N$, where the transition rates are modified by the conjugate variable s:

$$[\tilde{\mathbf{W}}(s)]_{ij} = k_{ij}e^{s\cdot\theta_{ij}} - \delta_{i,j}\sum_l k_{il}. \tag{S61}$$

Here, $\theta_{ij}\in\{+1,-1,0\}$ is the counting indicator for the transition $i\to j$. For example, to compute the statistics of the net mechanical flux $\psi = (N_{25}-N_{52})/t$, one specifically sets the indicators for the mechanical transitions as $\theta_{25} = +1$ and $\theta_{52} = -1$, while setting all other $\theta_{ij} = 0$. The scaled variance is then obtained from the second derivative of the largest real eigenvalue $\lambda(s)$ of $\tilde{\mathbf{W}}(s)$ at $s = 0$, $\lim_{t\to\infty} t\,\text{Var}[\psi] = \lambda''(0)$.

To compute the *gradient* vector $\nabla_{\mathbf{k}}r$, one requires the sensitivity $\partial\lambda''(0)/\partial k_{uv}$ for every rate constant. Using finite differences, this necessitates re-evaluating the eigenvalue $\lambda(s)$ (and its numerical derivatives) for each perturbed parameter.

- **Setup:** The cost is negligible ($\mathcal{T}_{\text{setup}}\approx 0$).
- **Step:** The bottleneck is the eigensolver. For a dense non-symmetric matrix, the cost scales as $\mathcal{T}_{\text{step}}\approx\mathcal{O}(N^3)$.

Substituting these into the total time yields:

$$\mathcal{T}_{\text{Koza}}\approx 0 + (2M)\,\mathcal{O}(N^3) = \mathcal{O}(M\cdot N^3). \tag{S62}$$

2. Algebraic RIM Method

Our method shifts the computational burden to constructing and inverting the Jacobian matrix $\mathbf{A}$. The dimension of $\mathbf{A}$ is determined by the edge count, $D_{\mathbf{A}} = 2M$.

- **Setup:** This is the bottleneck. Although $\mathbf{A}$ is sparse, its inverse $\mathbf{A}^{-1}$ is generally dense. Standard dense inversion scales cubically with the dimension: $\mathcal{T}_{\text{setup}} \approx \mathcal{O}(M^3)$.
- **Step:** Once $\mathbf{A}^{-1}$ is known, the gradient for each parameter involves multiplying specific matrix columns by the sparse derivative vector $\partial_{\mathbf{k}}\mathbf{A}$. Since $\partial_{\mathbf{k}}\mathbf{A}$ contains only $\mathcal{O}(1)$ non-zero elements, this vector-matrix product scales linearly with the matrix dimension: $\mathcal{T}_{\text{step}} \approx \mathcal{O}(M)$.

Substituting these into the total time yields:

$$\mathcal{T}_{\text{RIM}} \approx \mathcal{O}(M^3) + (2M) \cdot \mathcal{O}(M) = \mathcal{O}(M^3) + \mathcal{O}(M^2). \quad \text{(S63)}$$

Note that the iterative term is sub-dominant ($\mathcal{O}(M^2)$) compared to the setup term ($\mathcal{O}(M^3)$).

3. Scaling Comparison

The relative performance depends on the network topology. We analyze two distinct limits:

1. **Sparse / Physical Networks:** Biophysical models typically have localized connectivity, so $M \approx \mathcal{O}(N)$.

$$\mathcal{T}_{\text{Koza}} \sim (N) \cdot N^3 = \mathcal{O}(N^4) \quad \text{(S64)}$$

$$\mathcal{T}_{\text{RIM}} \sim (N)^3 + (N)^2 = \mathcal{O}(N^3) + \mathcal{O}(N^2) \quad \text{(S65)}$$

RIM achieves an asymptotic speed-up of factor N. The overhead of inverting a larger matrix ($2N \times 2N$) is outweighed by avoiding N repetitions of the eigensolver.

2. **Dense Networks ($M \approx \mathcal{O}(N^2)$:** In fully connected graphs, the number of edges grows quadratically.

$$\mathcal{T}_{\text{Koza}} \sim (N^2) \cdot N^3 = \mathcal{O}(N^5) \quad \text{(S66)}$$

$$\mathcal{T}_{\text{RIM}} \sim (N^2)^3 + (N^2)^2 = \mathcal{O}(N^6) + \mathcal{O}(N^4) \quad \text{(S67)}$$

The scaling advantage inverts. For extremely dense graphs, Koza's method is asymptotically superior because the dimension of the RIM Jacobian (N^2) grows much faster than the spectral matrix (N).

Note on Implementation. While $\mathbf{A}$ is a sparse matrix, its inverse $\mathbf{A}^{-1}$ is generally dense due to the global coupling of states in the NESS. Therefore, we utilize optimized dense linear algebra routines (e.g., LAPACK via NumPy) for the inversion. The scalability advantage of our method arises primarily from algebraic closure—computing the gradient requires only *one* matrix inversion and matrix-vector multiplications—rather than from the use of sparse inversion algorithms. This avoids the $2M$ repeated eigensolver calls required by generating function methods.

Note on Empirical Scaling. The complexity bounds derived above assume standard dense linear algebra scaling (e.g., cubic scaling for inversion and diagonalization). In practice, highly optimized numerical routines (such as LAPACK) often achieve better effective scaling (typically $\sim D^{2.4-2.8}$) for the intermediate matrix sizes models ($N \lesssim 100$). Consequently, while the *relative* ranking of the two methods remains consistent with our derivation, the absolute empirical exponents observed in numerical benchmarks may be lower than the worst-case theoretical bounds.

C. Numerical Benchmark: Extended Kinesin Model

To empirically validate the scalability of our approach, we benchmarked both methods using a generalized Kinesin network model. This setup corresponds to the results presented in Fig. 3 of the main text and utilizes the provided Python code.

1. Constructing the Extended Model

We utilized a "Θ-shape" topology (Fig. 3a in Main Text), which extends the standard 6-state Kinesin model [2] to arbitrary system sizes. The network consists of two fixed chemical branches (ATP-driven forward and backward cycles) connected by a central mechanical bridge. To vary the state-space size N_{sys}, we subdivided the single mechanical step ($2 \leftrightarrow 5$ in the original model) into a linear chain of N_{sub} mechanical substeps.

- **Topology:** The system consists of $N_{\text{sys}} = 4 + (N_{\text{sub}} + 1)$ states. The number of edges scales linearly with the system size, $M \approx 3N_{\text{sys}}$, representing a sparse physical topology typical of molecular motors.
- **Thermodynamic Consistency:** To ensure the model remains physically meaningful as N_{sub} increases, we must preserve the total chemical potential difference (cycle affinity) across the entire loop. The generalized detailed balance condition requires that the product of forward rates divided by the product of backward rates around the cycle satisfies:

$$\underbrace{\left(\frac{k^+_{\text{mech}}}{k^-_{\text{mech}}}\right)}_{\text{Mechanical}} \cdot \underbrace{\prod_{\text{chem}} \left(\frac{k^+_{\text{chem}}}{k^-_{\text{chem}}}\right)}_{\text{Chemical}} = \exp\left(\frac{\Delta\mu_{\text{ATP}} - F_{\text{load}} d}{k_B T}\right), \tag{S68}$$

  where the chemical ratio includes contributions from ATP binding, hydrolysis, and ADP release. If the mechanical bridge is divided into N_{sub} identical steps, the mechanical ratio becomes $(k^+_{\text{sub}}/k^-_{\text{sub}})^{N_{\text{sub}}}$. Therefore, to maintain the original driving force $\Delta\mu$, we follow Wagoner and Dill [3] and scale the individual substep rates geometrically:

$$k^{\pm}_{\text{sub}} = \left(k^{\pm}_{\text{total}}\right)^{1/N_{\text{sub}}}. \tag{S69}$$

  This ensures that the free energy drop per substep is exactly $\Delta G_{\text{total}}/N_{\text{sub}}$.
- **Numerical Stability:** To prevent artificial degeneracies in the linear solver arising from translational symmetry in the uniform chain, we applied a small random perturbation ($\pm 1\%$) to all transition rates.

2. Scaling Results

We computed the full gradient of the randomness parameter r with respect to all edge rates for system sizes ranging from $N_{\text{sub}} = 10$ to 320. The computation times are plotted in Fig. 3b of the main text.

1. **Koza's Method** (Red, $\approx N^{3.1}$): The brute-force spectral method requires iterating over all $2M$ parameters. For each parameter, it solves the eigenvalue problem for a sparse tilted matrix of size N_{sys}. The observed scaling exponent of 3.1 is consistent with the theoretical prediction: it is the product of the number of parameters ($N_{\text{param}} \propto N$) and the effective cost of the sparse eigensolver ($\mathcal{T}_{\text{eig}} \propto N^{2.1}$). Note that while the theoretical worst-case for eigensolvers is N^3, optimized solvers (like ARPACK) typically achieve effective scaling closer to $N^{2.1}$ for banded matrices of this size.
2. **Algebraic RIM Method** (Blue, $\approx N^{1.6}$): Our method performs a single matrix inversion of size $2M \times 2M$. The observed scaling exponent of 1.6 is remarkably low. This indicates that for the intermediate matrix sizes benchmarked here ($D_{\mathbf{A}} \lesssim 1000$), the direct linear solver operates in a highly efficient pre-asymptotic regime, scaling significantly better than the theoretical cubic bound (N^3). Crucially, the scaling gap ($3.1 - 1.6 = 1.5$) is even larger than the conservative theoretical prediction of 1.0 derived in the previous section. This effectively demonstrates that the "setup overhead" of RIM (inverting a larger matrix) is negligible compared to the "iterative penalty" of the spectral method.

For a system size of $N_{\text{sys}} \approx 100$, our method achieves a speed-up of over two orders of magnitude ($> 100\times$), confirming its practical utility for optimizing large-scale biophysical models.

D. Numerical Benchmark: Dense All-to-All Networks

To probe the performance limits of our edge-centric formalism, we benchmarked a fully connected network where every state is connected to every other state. This "all-to-all" topology ($M = N(N-1) \approx N^2$) represents the worst-case scenario for our approach, as the Jacobian dimension grows quadratically with the system size.

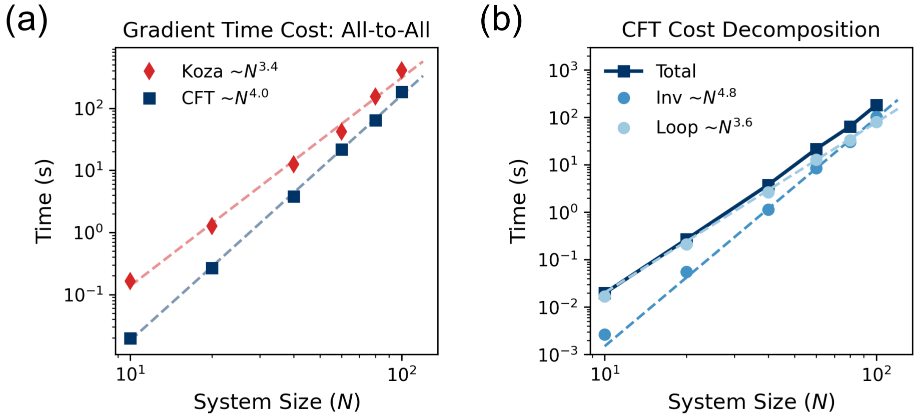

Figure S3. **Benchmarking on Dense All-to-All Networks.** **(a)** Comparison of total computation time between the Spectral method (Koza, red) and the Algebraic method (RIM, blue). Despite a lower asymptotic scaling exponent, Koza's method remains slower for $N \leq 100$ due to large prefactors. **(b)** Component breakdown of the RIM method. For large N, the matrix inversion cost (Setup, medium blue) dominates with a scaling of $\approx N^{4.8}$, while the gradient assembly loop (Loop, light blue) scales as $\approx N^{3.6}$.

1. *Scaling Results*

We computed the gradient computation time for system sizes up to $N = 100$. The results, shown in Fig. S3a and decomposed in Fig. S3b, reveal a performance crossover driven by the massive growth in connectivity.

1. **Koza's Method** (Red, $\approx N^{3.4}$): The number of rate parameters grows quadratically as $N_{\text{param}} \approx 2N^2$. Consequently, the method requires solving the $N \times N$ eigenvalue problem $2N^2$ times. The observed scaling ($N^{3.4}$) is significantly better than the theoretical worst-case bound ($N^2 \times N^3 = N^5$). This suggests that for the small dense matrices examined here ($N \leq 100$), the eigensolver operates in a pre-asymptotic regime with an effective scaling closer to $N^{1.4}$ per call. However, despite the favorable exponent, the absolute computation time is substantial due to the sheer number of repeated solver calls.

2. **Algebraic RIM Method** (Blue, $\approx N^{4.0}$): The Jacobian matrix dimension expands to $2N^2 \times 2N^2$. The total time scales as $N^{4.0}$, which is notably faster than the theoretical dense inversion scaling of $(N^2)^3 = N^6$. Decomposing the cost (Fig. S3b) clarifies this behavior:

   - **Inverse Cost (Setup):** Scales as $\approx N^{4.8}$. Since the matrix dimension is $D \sim N^2$, this corresponds to an effective solver scaling of $D^{2.4}$, which is consistent with optimized dense linear algebra routines (e.g., LAPACK/BLAS) on modern hardware. For sufficiently large N, this term will eventually dominate.
   - **Loop Cost (Gradient Assembly):** Scales as $\approx N^{3.6}$. This step involves iterating over $2N^2$ parameters and performing vector operations of size $2N^2$. The theoretical cost is $M^2 \sim N^4$, which closely matches the observed $N^{3.6}$.

   In the benchmarked range ($N \leq 100$), the Loop cost contributes significantly to the total time, weighing down the steeper scaling of the Inverse.

2. *The Crossover Argument*

Although Koza's method possesses a superior asymptotic exponent (3.4 vs 4.0) for dense graphs, **RIM remains computationally faster for system sizes up to $N \approx 100$.** This crossover behavior arises from the distinct computational overheads of the two approaches:

- **Koza (Cumulative Overhead):** This approach solves "Many Small Problems." The total time is $2N^2 \times T_{\text{small}}$. The repeated overhead of function calls, memory allocation, and convergence checking for the iterative eigensolver creates a large cumulative prefactor.

- **RIM (Vectorization Efficiency):** This approach solves "One Big Problem." The total time is $1 \times T_{\text{large}} + \text{fast loop}$. Modern CPUs are exceptionally efficient at handling single large matrix operations through cache optimization and vectorization.

Thus, even in the dense regime where our edge-based formalism is theoretically disadvantaged, the algebraic closure method remains a practical and competitive tool for exploring the design space of moderately sized, highly connected kinetic networks.

V. DETAILS OF RESULT 3: UNIFYING FLUCTUATION-RESPONSE RELATIONS

In this section, we provide the detailed derivation of the three classes of response relations presented in Result 3 of the main text. We show that they are all mathematical consequences of the fundamental identity $\mathbf{A}^{-1}\mathbf{A} = \mathbf{I}$. Furthermore, we demonstrate how these relations unify and generalize and some recent results in the literature [17, 18, 20] by extending them to arbitrary observables and simplifying their algebraic bounds.

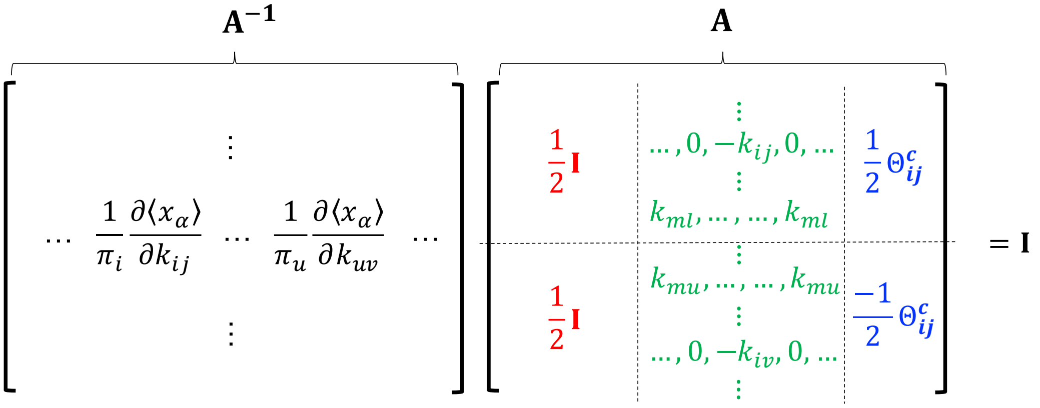

Figure S4. **Deriving Response Relations from Matrix Identity.** The Jacobian $\mathbf{A}$ (right) is structured into three blocks of columns. Multiplying the sensitivity vector (a row of $\mathbf{A}^{-1}$ associated with an arbitrary observable x) by these columns generates three distinct classes of constraints: Edge Reciprocity, Node Escaping Symmetry, and Cycle Symmetry.

A. Derivation of the Three Sets of Local Response Relations

As illustrated in Fig. S4, the Jacobian matrix $\mathbf{A}$ is constructed using a specific basis corresponding to the fundamental observables: symmetric edge traffic ϕ_{ij} (with mean $\langle \phi_{ij} \rangle = \tau_{ij}$), node occupation f_n (with mean $\langle f_n \rangle = \pi_n$), and cycle flux ψ_c (with mean $\langle \psi_c \rangle = J_c$). The fundamental identity $\mathbf{A}^{-1}\mathbf{A} = \mathbf{I}$ implies $\sum_\gamma [\mathbf{A}^{-1}]_{x,\gamma}[\mathbf{A}]_{\gamma,\beta} = \delta_{x,\beta}$. This generates three sets of constraints depending on the choice of the observable column β.

1. *Class I: Edge Reciprocity*

Selecting the column $\beta = \phi_{ij}$ corresponding to the symmetric traffic on edge (ij), the identity yields:

$$\frac{1}{\pi_i}\frac{\partial \langle x \rangle}{\partial k_{ij}} + \frac{1}{\pi_j}\frac{\partial \langle x \rangle}{\partial k_{ji}} = 2\delta_{\langle x \rangle, \tau_{ij}}. \tag{S70}$$

This relation dictates that for any observable x distinct from the local traffic τ_{ij} (e.g., a cycle flux J_c or state probability π_n), the response to the forward rate and backward rate must be equal and opposite (weighted by π). This symmetry is the engine allowing for the simplification of kinetic bounds discussed below.

2. Class II: Node Escaping Symmetry

Selecting the column $\beta = f_n$ corresponding to the frequency of node n, the identity yields:

$$\sum_{u(\neq m)} \frac{k_{mu}}{\pi_m} \frac{\partial\langle x\rangle}{\partial k_{mu}} - \sum_{l(\neq n)} \frac{k_{nl}}{\pi_n} \frac{\partial\langle x\rangle}{\partial k_{nl}} = \delta_{\langle x\rangle,\pi_n}. \tag{S71}$$

This describes the conservation of "sensitivity flux" at each node. It states that the sum of normalized sensitivities for rates entering a reference node m differs from those leaving node n only if the observable is the node occupancy π_n itself.

3. Class III: Cycle Symmetry

Selecting the column $\beta = \psi_c$ corresponding to a fundamental cycle flux, the identity yields:

$$\sum_{(ij)\in c^+} \frac{1}{\pi_i} \frac{\partial\langle x\rangle}{\partial k_{ij}} - \sum_{(ji)\in c^-} \frac{1}{\pi_j} \frac{\partial\langle x\rangle}{\partial k_{ji}} = 2\delta_{\langle x\rangle,J_c}. \tag{S72}$$

This shows that the integrated sensitivity along a forward cycle must balance that of the backward cycle, with a net residue appearing only if the observable is the cycle flux J_c itself.

B. Generalizing Owen *et al.* [17] via Node Escaping Symmetry

The "universal stoichiometric response" derived by Owen *et al.* [17] describes how state probabilities respond to perturbations. Their central result is:

$$\sum_{j(\neq i)} k_{ij} \frac{\partial \pi_n}{\partial k_{ij}} = -\delta_{in}\pi_n(1-\pi_n) + (1-\delta_{in})\,\pi_i\pi_n. \tag{S73}$$

In this section, we show that this result is a specific instance of our **Node Escaping Symmetry**. Our framework is more general because the Node Escaping Symmetry applies to *any* NESS observable (including traffic and cycle fluxes), whereas Eq. (S73) is the specific projection of this symmetry onto state probabilities, constrained by normalization.

Step 1: The Node Energy Shorthand

We first identify the physical meaning of the summation operator on the left-hand side of Eq. (S73). Consider a parameterization where each node has an "energy" E_i such that $k_{ij} \propto e^{E_i}$. Physically, perturbing E_i corresponds to changing the "energy" of state i while fixing all other landscape dimensions (barriers and other state energies). Mathematically, this provides a shorthand for the aggregate sensitivity:

$$\frac{\partial \pi_n}{\partial E_i} \equiv \sum_{j(\neq i)} \frac{\partial \pi_n}{\partial k_{ij}} \frac{\partial k_{ij}}{\partial E_i} = \sum_{j(\neq i)} k_{ij} \frac{\partial \pi_n}{\partial k_{ij}}. \tag{S74}$$

Step 2: Applying the Node Escaping Symmetry

We now apply our Node Escaping Symmetry (Eq. S71) to the observable $\langle x\rangle = \pi_n$. The symmetry relates the sensitivity flux originating from a reference node m to that of an arbitrary node i:

$$\sum_{u(\neq m)} \frac{k_{mu}}{\pi_m} \frac{\partial \pi_n}{\partial k_{mu}} - \sum_{j(\neq i)} \frac{k_{ij}}{\pi_i} \frac{\partial \pi_n}{\partial k_{ij}} = \delta_{in} - \delta_{mn}. \tag{S75}$$

Using shorthand $\partial_{E_i}\pi_n = \sum_{j\neq i} k_{ij}\partial_{k_{ij}}\pi_n$, we get:

$$\frac{1}{\pi_m}\frac{\partial \pi_n}{\partial E_m} - \frac{1}{\pi_i}\frac{\partial \pi_n}{\partial E_i} = \delta_{in} - \delta_{mn}. \tag{S76}$$

To isolate the dependence on node i, we rearrange the terms, grouping all i-dependent terms on the left and m-dependent terms on the right:

$$\frac{1}{\pi_i}\frac{\partial \pi_n}{\partial E_i} + \delta_{in} = \frac{1}{\pi_m}\frac{\partial \pi_n}{\partial E_m} + \delta_{mn}. \tag{S77}$$

Since the right-hand side depends only on the fixed reference node m (and the target state n), while the left-hand side depends on the arbitrary source node i, both sides must be equal to a quantity C_n that is *constant* with respect to i. Thus, the normalized response must follow the structural form:

$$\frac{1}{\pi_i}\frac{\partial \pi_n}{\partial E_i} = C_n - \delta_{in}, \tag{S78}$$

where C_n is a constant determined by the reference term.

Step 3: Determining the Constant via Homogeneity

To determine C_n, we invoke the time-scale invariance of Markov processes. Since steady-state probabilities π_n depend only on rate ratios, they are homogeneous functions of degree 0 with respect to the rates. This implies that a uniform shift in all node energies ($E_i \to E_i + \delta E$), which scales all rates globally, leaves π_n unchanged:

$$\sum_{\text{all } i} \frac{\partial \pi_n}{\partial E_i} = 0. \tag{S79}$$

Substituting our structural form $\partial_{E_i}\pi_n = \pi_i(C_n - \delta_{in})$ into this constraint:

$$\begin{aligned} &\sum_i \pi_i(C_n - \delta_{in}) = 0 \\ &C_n \underbrace{\sum_i \pi_i}_{1} - \underbrace{\sum_i \pi_i \delta_{in}}_{\pi_n} = 0 \quad \Rightarrow \quad C_n = \pi_n. \end{aligned} \tag{S80}$$

Step 4: Final Result

With $C_n = \pi_n$, Eq. (S78) becomes:

$$\frac{1}{\pi_i}\frac{\partial \pi_n}{\partial E_i} = \pi_n - \delta_{in}. \tag{S81}$$

Multiplying by π_i and expanding the shorthand ∂_{E_i}, we recover:

$$\sum_{j(\neq i)} k_{ij}\frac{\partial \pi_n}{\partial k_{ij}} = \pi_i\pi_n - \pi_i\delta_{in}. \tag{S82}$$

This matches Eq. (S73) exactly.

Conclusion: This derivation confirms that the stoichiometric response is not an isolated identity but a consequence of the Node Escaping Symmetry. Crucially, while Euler's homogeneity fixes the constant C_n specifically for probabilities, the underlying symmetry structure (Eq. S78) remains valid for *any* observable, such as cycle fluxes or edge traffic. We next show how this leads to new response equalities.

C. How Traffic and Cycle Flux response to escaping rate tuning.

We derive here that for traffic τ_{ij} or cycle flux J_c (or their linear combinations), their responses to tuning one node's escaping rate (i.e. E_n) have a simple expression:

$$\frac{\partial \tau_{ij}}{\partial E_n} = \sum_{l(\neq n)} k_{nl} \frac{\partial \tau_{ij}}{\partial k_{nl}} = \pi_n \tau_{ij}; \tag{S83}$$

$$\frac{\partial J_c}{\partial E_n} = \sum_{l(\neq n)} k_{nl} \frac{\partial J_c}{\partial k_{nl}} = \pi_n J_c; \tag{S84}$$

Step 1: The Symmetry Condition

For any flow observable $\langle y\rangle \notin \{\pi_n\}$ (where $\delta_{\langle y\rangle,\pi_n} = 0$), substituting the energy perturbation operator defined in Eq. (S74), $\partial_{E_n} \equiv \sum_{j(\neq n)} k_{nj}\partial_{k_{nj}}$, the symmetry relation in Eq. (S71) simplifies to:

$$\frac{1}{\pi_m}\frac{\partial\langle y\rangle}{\partial E_m} - \frac{1}{\pi_n}\frac{\partial\langle y\rangle}{\partial E_n} = 0. \tag{S85}$$

This implies that the sensitivity, when normalized by the local population, is spatially uniform across the network:

$$\frac{\partial\langle y\rangle}{\partial E_n} = C\pi_n, \tag{S86}$$

where C is a constant specific to the observable $\langle y\rangle$ but independent of the node index n.

Step 2: Determining the Constant via Homogeneity

To determine C, we again invoke the time-scale invariance. Unlike the probabilities π (which are degree 0), fluxes and traffic rates have physical units of inverse time (t^{-1}). Consequently, they are homogeneous functions of *degree 1* with respect to the transition rates. A global shift in energy $E_n \to E_n + \delta E$ scales all rates by $e^{\delta E}$, and thus must scale the total magnitude of flow variables $\langle y\rangle$ by $e^{\delta E}$ without altering relative branching. Euler's homogeneous function theorem dictates:

$$\sum_n \frac{\partial\langle y\rangle}{\partial E_n} = \langle y\rangle. \tag{S87}$$

Substituting the structural form from Eq. (S86) into this constraint:

$$\sum_n (C\pi_n) = C\underbrace{\sum_n \pi_n}_{1} = \langle y\rangle \quad \Longrightarrow \quad C = \langle y\rangle. \tag{S88}$$

Step 3: The Scaling Law

We thus arrive at the universal scaling relation for any steady-state flow observable $\langle y\rangle \in \{\tau_{ij}, J_c\}$:

$$\frac{\partial\langle y\rangle}{\partial E_n} = \pi_n\langle y\rangle. \tag{S89}$$

This result proves that the perturbation of escaping rates (defined by tuning E_n) are degenerate controls for flux routing: they cannot alter the relative ratios of fluxes (pathways) in a network, but rather act locally as probability-weighted accelerators that scale the global magnitude of the flow.

D. Barrier Tuning and the Net Flux Constraint

We now consider the response to tuning "the barrier height B_{ij}" of a transition. Physically, raising a barrier decreases both the forward and backward rates simultaneously. We define the barrier perturbation operator as:

$$\frac{\partial}{\partial B_{ij}} \equiv -k_{ij}\frac{\partial}{\partial k_{ij}} - k_{ji}\frac{\partial}{\partial k_{ji}}. \tag{S90}$$

We show here that barrier tuning is governed by a strict Net Flux Constraint: it cannot affect any global observable $\langle x\rangle$ (except the local traffic τ_{ij}) unless the edge carries a non-zero net flux ($J_{ij} \neq 0$).

For any observable $\langle x\rangle \neq \tau_{ij}$ (e.g., state probabilities π_n, cycle fluxes J_c, or other edge traffic τ_{uv}, $uv \neq ij$), the right-hand side of the Edge Reciprocity relation (Eq. S70) vanishes:

$$\frac{1}{\pi_i}\frac{\partial\langle x\rangle}{\partial k_{ij}} + \frac{1}{\pi_j}\frac{\partial\langle x\rangle}{\partial k_{ji}} = 0. \tag{S91}$$

This enforces a strict coupling between the forward and backward rate sensitivities:

$$\frac{\partial\langle x\rangle}{\partial k_{ji}} = -\frac{\pi_j}{\pi_i}\frac{\partial\langle x\rangle}{\partial k_{ij}}. \tag{S92}$$

Substituting this into the barrier operator:

$$\begin{aligned}\frac{\partial\langle x\rangle}{\partial B_{ij}} &= -k_{ij}\frac{\partial\langle x\rangle}{\partial k_{ij}} - k_{ji}\left(-\frac{\pi_j}{\pi_i}\frac{\partial\langle x\rangle}{\partial k_{ij}}\right)\\ &= -\left(k_{ij} - k_{ji}\frac{\pi_j}{\pi_i}\right)\frac{\partial\langle x\rangle}{\partial k_{ij}}\\ &= -\frac{1}{\pi_i}\underbrace{(\pi_i k_{ij} - \pi_j k_{ji})}_{J_{ij}}\frac{\partial\langle x\rangle}{\partial k_{ij}}.\end{aligned} \tag{S93}$$

This yields the explicit response formula:

$$\frac{\partial\langle x\rangle}{\partial B_{ij}} = -\frac{J_{ij}}{\pi_i}\frac{\partial\langle x\rangle}{\partial k_{ij}}. \tag{S94}$$

Conclusion: If the edge is detailed-balanced ($J_{ij} = 0$), the response vanishes ($\partial_{B_{ij}}\langle x\rangle = 0$). This proves that barrier perturbations cannot tune $\langle x\rangle \neq \tau_{ij}$ if the edge is "locally" at equilibrium, $J_{ij} = 0$. They acquire control authority over global landscape properties (like π and J_c) only through the local net flux J_{ij} generated by nonequilibrium driving.

E. Generalizing the Flux Covariance from Aslyamov *et al.* [20]

Aslyamov *et al.* [20] presented a decomposition for the covariance of cycle fluxes. We show this is a direct consequence of our Edge Reciprocity and covariance parsing. The covariance decomposition (Result 1) is:

$$t\cdot \mathrm{CoV}[\psi_c,\psi_{c'}] = \sum_{i<j}\left(\frac{k_{ij}}{\pi_i}\frac{\partial J_c}{\partial k_{ij}}\frac{\partial J_{c'}}{\partial k_{ij}} + \frac{k_{ji}}{\pi_j}\frac{\partial J_c}{\partial k_{ji}}\frac{\partial J_{c'}}{\partial k_{ji}}\right). \tag{S95}$$

Following Owen *et al.* [17] and Aslyamov *et al.* [20], one can consider the parameterization $k_{ij} = \exp[E_i - B_{ij} + F_{ij}/2]$ to consider various types of rate perturbations.

1. Barrier Sensitivity (∂B_{ij})

Perturbing the symmetric barrier parameter B_{ij} leads to $\frac{\partial}{\partial B_{ij}} := -k_{ij}\frac{\partial}{\partial k_{ij}} - k_{ji}\frac{\partial}{\partial k_{ji}}$. Since J_c is not a local traffic variable, Eq. (S70) implies the reciprocity:

$$\frac{1}{\pi_i}\frac{\partial J_c}{\partial k_{ij}} = -\frac{1}{\pi_j}\frac{\partial J_c}{\partial k_{ji}}. \tag{S96}$$

This allows us to equate the rate sensitivity solely to the barrier sensitivity:

$$\frac{\partial J_c}{\partial B_{ij}} = -\left(k_{ij} - k_{ji}\frac{\pi_j}{\pi_i}\right)\frac{\partial J_c}{\partial k_{ij}} = -\frac{J_{ij}}{\pi_i}\frac{\partial J_c}{\partial k_{ij}} \quad \Rightarrow \quad \frac{\partial J_c}{\partial k_{ij}} = -\frac{\pi_i}{J_{ij}}\frac{\partial J_c}{\partial B_{ij}}. \tag{S97}$$

Now, we substitute the reciprocity relation Eq. (S96) back into the covariance sum in Eq. (S95). The term inside the parenthesis for a given edge ij becomes:

$$\begin{aligned} &\frac{k_{ij}}{\pi_i}\frac{\partial J_c}{\partial k_{ij}}\frac{\partial J_{c'}}{\partial k_{ij}} + \frac{k_{ji}}{\pi_j}\left(-\frac{\pi_j}{\pi_i}\frac{\partial J_c}{\partial k_{ij}}\right)\left(-\frac{\pi_j}{\pi_i}\frac{\partial J_{c'}}{\partial k_{ij}}\right) \\ &= \left(\frac{k_{ij}}{\pi_i} + \frac{k_{ji}\pi_j}{\pi_i^2}\right)\frac{\partial J_c}{\partial k_{ij}}\frac{\partial J_{c'}}{\partial k_{ij}} \\ &= \frac{1}{\pi_i^2}\underbrace{(\pi_i k_{ij} + \pi_j k_{ji})}_{\tau_{ij}}\frac{\partial J_c}{\partial k_{ij}}\frac{\partial J_{c'}}{\partial k_{ij}}. \end{aligned} \tag{S98}$$

Finally, plugging in the barrier sensitivity from Eq. (S97), we obtain:

$$\begin{aligned} \dots &= \frac{\tau_{ij}}{\pi_i^2}\left(-\frac{\pi_i}{J_{ij}}\frac{\partial J_c}{\partial B_{ij}}\right)\left(-\frac{\pi_i}{J_{ij}}\frac{\partial J_{c'}}{\partial B_{ij}}\right) \\ &= \frac{\tau_{ij}}{J_{ij}^2}\frac{\partial J_c}{\partial B_{ij}}\frac{\partial J_{c'}}{\partial B_{ij}}. \end{aligned} \tag{S99}$$

Summing over all edges $i < j$ recovers the first result from Aslyamov *et al.* [20]:

$$t \cdot \mathrm{CoV}[\psi_c, \psi_{c'}] = \sum_{i<j}\frac{\tau_{ij}}{J_{ij}^2}\frac{\partial J_c}{\partial B_{ij}}\frac{\partial J_{c'}}{\partial B_{ij}}. \tag{S100}$$

2. *Force Sensitivity* (∂F_{ij})

In Aslyamov *et al.* [20], the covariance is also expanded by the responses to the driving force F_{ij}, defined by $\partial_{F_{ij}} := \frac{1}{2}\left(k_{ij}\partial_{k_{ij}} - k_{ji}\partial_{k_{ji}}\right)$. Using the reciprocity relation in Eq. (S96) to express $\partial_{k_{ji}}$ in terms of $\partial_{k_{ij}}$, the force sensitivity simplifies to:

$$\begin{aligned} \frac{\partial J_c}{\partial F_{ij}} &= \frac{1}{2}\left(k_{ij}\frac{\partial J_c}{\partial k_{ij}} - k_{ji}\left(-\frac{\pi_j}{\pi_i}\frac{\partial J_c}{\partial k_{ij}}\right)\right) \\ &= \frac{1}{2\pi_i}\underbrace{(\pi_i k_{ij} + \pi_j k_{ji})}_{\tau_{ij}}\frac{\partial J_c}{\partial k_{ij}} = \frac{\tau_{ij}}{2\pi_i}\frac{\partial J_c}{\partial k_{ij}}. \end{aligned} \tag{S101}$$

Inverting this relation gives the rate sensitivity explicitly in terms of the force sensitivity:

$$\frac{\partial J_c}{\partial k_{ij}} = \frac{2\pi_i}{\tau_{ij}}\frac{\partial J_c}{\partial F_{ij}}. \tag{S102}$$

We substitute this back into the traffic-form of the covariance sum derived in Eq. (S98). The term for edge ij becomes:

$$\begin{aligned} \frac{\tau_{ij}}{\pi_i^2}\left(\frac{\partial J_c}{\partial k_{ij}}\right)\left(\frac{\partial J_{c'}}{\partial k_{ij}}\right) &= \frac{\tau_{ij}}{\pi_i^2}\left(\frac{2\pi_i}{\tau_{ij}}\frac{\partial J_c}{\partial F_{ij}}\right)\left(\frac{2\pi_i}{\tau_{ij}}\frac{\partial J_{c'}}{\partial F_{ij}}\right) \\ &= \frac{\tau_{ij}}{\pi_i^2}\left(\frac{4\pi_i^2}{\tau_{ij}^2}\right)\frac{\partial J_c}{\partial F_{ij}}\frac{\partial J_{c'}}{\partial F_{ij}} \\ &= \frac{4}{\tau_{ij}}\frac{\partial J_c}{\partial F_{ij}}\frac{\partial J_{c'}}{\partial F_{ij}}. \end{aligned} \tag{S103}$$

Summing over all edges recovers the complementary expansion in Aslyamov *et al.* [20]:

$$t \cdot \mathrm{CoV}[\psi_c, \psi_{c'}] = \sum_{i<j}\frac{4}{\tau_{ij}}\frac{\partial J_c}{\partial F_{ij}}\frac{\partial J_{c'}}{\partial F_{ij}}. \tag{S104}$$

Summary: Unifying the Decompositions

This derivation establishes that the flux covariance decompositions derived in Ref. [20] are specific instances of our general covariance parsing (Result 1). Our framework provides the overarching geometry: by restricting the general observables x and x' to cycle fluxes ($J_c, J_{c'}$) and applying our **Edge Reciprocity** to map the canonical rate sensitivities (∂_k) onto the specific barrier (∂_B) and force (∂_F) parameterizations, we exactly recover their identities. Consequently, our $\mathbf{A}^{-1}$ formalism generalizes these fluctuation-response dualities beyond net fluxes, making them applicable to the full set of nonequilibrium observables including traffic and state occupancies.

F. Translating the Kinetic Bounds from Aslyamov and Esposito [18]

Aslyamov and Esposito [18] derived bounds on the response to the parameter B_{ij}. They proved that the quantities $\Delta_{ij} \equiv 1 + \partial_{B_{ij}} \ln |J_{ij}|$ and $\nabla_{ij} \equiv (\tau_{ij}/J_{ij})(1 + \partial_{B_{ij}} \ln \tau_{ij})$ satisfy:

$$0 \le \Delta_{ij} \le 1 \quad \text{and} \quad |\nabla_{ij}| \le \Delta_{ij}. \tag{S105}$$

Our Edge Reciprocity translates these directly into simple inequalities for elementary rate sensitivities.

Step 1: Expressing Δ_{ij}.

Using the definition and the flux reciprocity (Eq. S97):

$$\begin{aligned}\Delta_{ij} &= 1 + \frac{1}{J_{ij}} \frac{\partial J_{ij}}{\partial B_{ij}} \\ &= 1 + \frac{1}{J_{ij}} \left(-\frac{J_{ij}}{\pi_i} \frac{\partial J_{ij}}{\partial k_{ij}} \right) \\ &= 1 - \frac{1}{\pi_i} \frac{\partial J_{ij}}{\partial k_{ij}}.\end{aligned} \tag{S106}$$

Step 2: Expressing ∇_{ij}.

We apply the Edge Reciprocity to the traffic observable τ_{ij}. Since τ_{ij} has a non-zero self-response ($2\delta = 2$), Eq. (S70) gives $\frac{1}{\pi_i} \frac{\partial \tau_{ij}}{\partial k_{ij}} + \frac{1}{\pi_j} \frac{\partial \tau_{ij}}{\partial k_{ji}} = 2$. The sensitivity to B-perturbation is:

$$\begin{aligned}\frac{\partial \tau_{ij}}{\partial B_{ij}} &= -k_{ij} \frac{\partial \tau_{ij}}{\partial k_{ij}} - k_{ji} \frac{\partial \tau_{ij}}{\partial k_{ji}} \\ &= -k_{ij} \frac{\partial \tau_{ij}}{\partial k_{ij}} - k_{ji} \pi_j \left(\frac{2}{\pi_j} - \frac{1}{\pi_i} \frac{\partial \tau_{ij}}{\partial k_{ij}} \right) \\ &= -\left(k_{ij} - \frac{\pi_j k_{ji}}{\pi_i} \right) \frac{\partial \tau_{ij}}{\partial k_{ij}} - 2k_{ji}\pi_j \\ &= -\frac{J_{ij}}{\pi_i} \frac{\partial \tau_{ij}}{\partial k_{ij}} - (\tau_{ij} - J_{ij}).\end{aligned} \tag{S107}$$

Substituting this into the definition of ∇_{ij}:

$$\begin{aligned}\nabla_{ij} &= \frac{\tau_{ij}}{J_{ij}} \left(1 + \frac{1}{\tau_{ij}} \frac{\partial \tau_{ij}}{\partial B_{ij}} \right) \\ &= \frac{\tau_{ij}}{J_{ij}} + \frac{1}{J_{ij}} \left(-\frac{J_{ij}}{\pi_i} \frac{\partial \tau_{ij}}{\partial k_{ij}} - \tau_{ij} + J_{ij} \right) \\ &= 1 - \frac{1}{\pi_i} \frac{\partial \tau_{ij}}{\partial k_{ij}}.\end{aligned} \tag{S108}$$

Step 3: Deriving the Hierarchy.

Substituting these expressions back into the Aslyamov bounds allows us to derive the kinetic hierarchy. We utilize the linear relations between CFT observables and one-way fluxes: $\tau_{ij} = p_{ij} + p_{ji}$ and $J_{ij} = p_{ij} - p_{ji}$. The derivatives satisfy:

$$\frac{\partial \tau_{ij}}{\partial k_{ij}} = \frac{\partial p_{ij}}{\partial k_{ij}} + \frac{\partial p_{ji}}{\partial k_{ij}} \quad \text{and} \quad \frac{\partial J_{ij}}{\partial k_{ij}} = \frac{\partial p_{ij}}{\partial k_{ij}} - \frac{\partial p_{ji}}{\partial k_{ij}}. \tag{S109}$$

We now expand the three inequalities implied by $|\nabla_{ij}| \leq \Delta_{ij}$ and $0 \leq \Delta_{ij} \leq 1$:

1. The Le Chatelier Bound ($\nabla_{ij} \leq \Delta_{ij}$):

$$\begin{aligned} 1 - \frac{1}{\pi_i}\frac{\partial \tau_{ij}}{\partial k_{ij}} &\leq 1 - \frac{1}{\pi_i}\frac{\partial J_{ij}}{\partial k_{ij}} \\ -\frac{\partial \tau_{ij}}{\partial k_{ij}} &\leq -\frac{\partial J_{ij}}{\partial k_{ij}} \implies \frac{\partial \tau_{ij}}{\partial k_{ij}} \geq \frac{\partial J_{ij}}{\partial k_{ij}}. \end{aligned} \tag{S110}$$

   Substituting the p-derivatives:

$$\begin{aligned} \left(\frac{\partial p_{ij}}{\partial k_{ij}} + \frac{\partial p_{ji}}{\partial k_{ij}}\right) &\geq \left(\frac{\partial p_{ij}}{\partial k_{ij}} - \frac{\partial p_{ji}}{\partial k_{ij}}\right) \\ 2\frac{\partial p_{ji}}{\partial k_{ij}} \geq 0 \quad &\implies \quad \frac{\partial p_{ji}}{\partial k_{ij}} \geq 0. \end{aligned} \tag{S111}$$

2. The Causality Bound ($\Delta_{ij} \leq 1$):

$$1 - \frac{1}{\pi_i}\frac{\partial J_{ij}}{\partial k_{ij}} \leq 1 \implies \frac{\partial J_{ij}}{\partial k_{ij}} \geq 0. \tag{S112}$$

   In terms of one-way fluxes, this yields:

$$\frac{\partial p_{ij}}{\partial k_{ij}} - \frac{\partial p_{ji}}{\partial k_{ij}} \geq 0 \quad \implies \quad \frac{\partial p_{ij}}{\partial k_{ij}} \geq \frac{\partial p_{ji}}{\partial k_{ij}}. \tag{S113}$$

3. The Population Depletion Bound ($-\Delta_{ij} \leq \nabla_{ij}$):

$$\begin{aligned} -\left(1 - \frac{1}{\pi_i}\frac{\partial J_{ij}}{\partial k_{ij}}\right) &\leq 1 - \frac{1}{\pi_i}\frac{\partial \tau_{ij}}{\partial k_{ij}} \\ -1 + \frac{1}{\pi_i}\frac{\partial J_{ij}}{\partial k_{ij}} &\leq 1 - \frac{1}{\pi_i}\frac{\partial \tau_{ij}}{\partial k_{ij}} \\ \frac{1}{\pi_i}\left(\frac{\partial J_{ij}}{\partial k_{ij}} + \frac{\partial \tau_{ij}}{\partial k_{ij}}\right) &\leq 2. \end{aligned} \tag{S114}$$

   Substituting the sum $\partial_k J + \partial_k \tau = 2\partial_k p_{ij}$:

$$\frac{1}{\pi_i}\left(2\frac{\partial p_{ij}}{\partial k_{ij}}\right) \leq 2 \quad \implies \quad \frac{\partial p_{ij}}{\partial k_{ij}} \leq \pi_i. \tag{S115}$$

Combining these three results yields the strict kinetic hierarchy presented in Eq. (5) of the Main Text:

$$\pi_i \geq \frac{\partial p_{ij}}{\partial k_{ij}} \geq \frac{\partial p_{ji}}{\partial k_{ij}} \geq 0. \tag{S116}$$
